# Measuring Zn Transference with Precision: Insights for Dendrite‐Free Zinc Metal Anodes

**DOI:** 10.1002/adma.202502245

**Published:** 2025-08-23

**Authors:** Dario Gomez Vazquez, Julita Tabor, Travis P. Pollard, Oleg Borodin, Maria R. Lukatskaya

**Affiliations:** ^1^ Department of Mechanical and Process Engineering ETH Zurich Zurich 8092 Switzerland; ^2^ DEVCOM Army Research Laboratory Battery Science Branch Energy Sciences Division Adelphi MD 20783 USA

**Keywords:** batteries, electrolytes, transference number, zinc

## Abstract

Electrolyte engineering in Zn‐metal batteries frequently employs alkaline metal salts to enhance conductivity and reduce overpotential for Zn plating. While these additives improve conductivity, the presence of more mobile alkali cations can negatively affect the Zn^2+^ transference number. Optimizing this property is crucial for high‐rate performance, efficiency, and safety, as high Zn^2+^ transference number minimizes concentration polarization and dendrite formation during high‐current cycling. However, reliably measuring the transference number in non‐binary electrolytes presents significant experimental challenges due to dynamic Zn metal interfaces, rendering traditional methods ineffective. Here, we use a modified Hittorf‐type method to measure Zn^2+^ transference numbers in complex electrolytes. Supported by molecular dynamics simulations, this method is applied to obtain transference numbers of Zn^2+^, K^+,^ and acetate ions in Zn‐K acetate electrolytes. By varying the Zn^2+^ fraction, the impact of co‐salts on transport properties is studied and correlated with the Zn solvation environment using X‐ray absorption spectroscopy. It is revealed that while ionic conductivity increases with the addition of KOAc co‐salt, the Zn^2+^ transference number dramatically decreases. Electrolytes with higher Zn^2+^ transference numbers enable longer high‐rate cycling, underscoring the importance of optimizing Zn^2+^ transference for improved performance of Zn‐metal anodes.

## Graphical abstract

A modified Hittorf method reveals how co‐salt composition affects Zn^2+^ transference numbers. Coupled with molecular dynamics and spectroscopy, we shows that electrolytes with high Zn transference number (tZn) suppress dendrites, enabling longer high‐rate cycling and more stable Zn‐metal anodes.

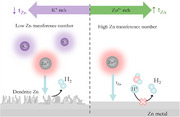

## 1 Introduction

Lithium‐ion batteries, despite their current dominance, face significant challenges for large‐scale, long‐term grid energy storage, including high cost, safety concerns, and environmental impact.^[^ ^1^, ^2^ ^]^ Aqueous batteries, particularly Zn metal batteries, emerge as a compelling alternative due to the high theoretical capacities of Zn metal anodes (≈820 mAh g^−1^ gravimetric and ≈5800 mAh cm^−3^ volumetric), their lower cost, enhanced safety, and sustainability.^[^ ^3^, ^4^ ^]^ However, the commercialization of Zn metal batteries is challenged by the dendrite formation during Zn plating and the concurrent hydrogen evolution reaction (HER).^[^ ^5^ ^]^ These processes reduce cycle life and compromise safety, ultimately leading to inefficient operation and shorter battery life.^[^ ^5^, ^6^ ^]^

Recently, electrolyte engineering emerged as an effective approach to improve the performance and longevity of Zn‐metal anodes. Many studied electrolyte formulations often incorporate alkali metal supporting salts (e.g., LiTFSI,^[^ ^7^ ^]^ KOAc,^[^ ^8^ ^]^ NaClO_4,_^[^ ^9^ ^]^ LiOTf^[^ ^10^ ^]^), which enhance ionic conductivity and therefore reduce the overpotential for Zn plating/stripping and/or can enable very high concentrations for water‐in‐salt (WiS) regimes^[^ ^7^, ^8^, ^9^, ^10^ ^]^ that help suppress HER. While these co‐salts can boost electrolyte conductivity,^[^ ^11^, ^12^ ^]^ they also introduce competing mobile cations which can negatively impact the Zn^2+^ transference number.^[^ ^13^, ^14^ ^]^

For Li metal batteries a clear correlation was demonstrated between high transference number of Li^+^ and improved high‐rate performance and cycling efficiency.^[^ ^15^, ^16^ ^]^ A high transference number minimizes concentration gradients during polarization, thus reducing charge/discharge overpotential and mitigating dendrite formation during high‐current cycling.^[^ ^15^, ^17^, ^18^, ^19^ ^]^ However, for Zn‐metal batteries, the effect of co‐salts on the Zn^2+^ transference number is less well understood, despite its crucial role in determining battery performance and stability. This can be explained by the fact that measuring the Zn^2+^ transference number in non‐binary electrolyte systems presents significant experimental challenges.^[^ ^20^, ^21^, ^22^, ^23^ ^]^ Traditional approaches, such as the Bruce‐Vincent method, fail to yield accurate transference numbers in complex multicomponent electrolytes due to the single‐ion quantification capabilities.^[^ ^15^, ^24^ ^]^ Moreover, the dynamic nature of Zn metal interfaces, make this approach suboptimal for robust transference number quantification.^[^ ^25^, ^26^ ^]^ Other methods such as pulse field gradient nuclear magnetic resonance (NMR) are not practical for Zn as its only NMR‐active ^67^Zn isotope is a low‐sensitivity nucleus with a strong quadrupolar moment requiring high fields and complex pulse sequences.^[^ ^27^, ^28^, ^29^, ^30^ ^]^ Because of the challenges associated with the transference number measurement in complex electrolytes, many studies often omit measuring it, instead drawing conclusions based solely on the electrolyte's viscosity and total ionic conductivity.^[^ ^31^, ^32^ ^]^ In other instances, studies employ single‐ion measurement techniques for transference number determination, and thus are unable to gain deeper insights from the complete set of transport properties of an electrolyte.^[^ ^33^, ^34^ ^]^ When the Bruce–Vincent method is used, reported t_Zn_ values vary widely even when measured for electrolyte with near‐identical composition—for example, t_Zn_ = 0.2 was reported for 30 m ZnCl_2_ aqueous electrolyte^[^ ^33^ ^]^ versus 0.7 for 31 m ZnCl_2_ aqueous electrolyte.^[^ ^34^ ^]^ This limitation underscores the need for a robust approach that can reliably quantify the transport properties of Zn^2+^ in the presence of co‐electrolytes.^[^ ^35^, ^36^ ^]^

In this work, we address these challenges by introducing a modified Hittorf‐type method for reliably measuring the Zn^2+^ transference number in complex, non‐binary concentrated electrolytes. This method enables us to investigate the effects of co‐electrolytes on the transport properties of Zn^2+^, K^+^, and acetate (OAc^−^) ions in multi‐component electrolytes. We select acetate salts due to their low cost, intrinsic safety, environmental friendliness, and minimal corrosion risk compared with halide‐based electrolytes.^[^ ^31^, ^37^ ^]^ Additionally, we employ X‐ray absorption spectroscopy (XAS) to correlate transport properties with the solvation environment of Zn^2+^, providing deeper insights into how co‐salts influence Zn^2+^ mobility and coordination. We then correlate our findings with the Zn plating/stripping efficiency, morphology, and cycling stability for different current densities.

## 2 Results and Discussion

### 2.1 Electrolyte Physicochemical Properties

In this work, we investigate the influence of co‐salt fractions on the solvation environment of Zn cations, transport properties, Zn deposition efficiency, and morphology. To achieve this, we examine a Zn_X_K_1−X_(OAc)_1+X_·30H_2_O electrolyte series (x = 0.2, 0.4, 0.6, 0.8, 1), systematically varying the Zn^2+^ fraction. By fixing the concentration at 30 H_2_O molecules per cation, we ensure a consistent basis for comparison, avoiding contributions from concentration‐induced changes in solvation environment and electrolyte properties.^[^ ^8^ ^]^ The 30 H_2_O per cation concentration was selected as it corresponds to the maximum solubility of Zn(OAc)_2_, allowing us to explore a broad range of Zn:K compositional ratios.

**Figure 1:**
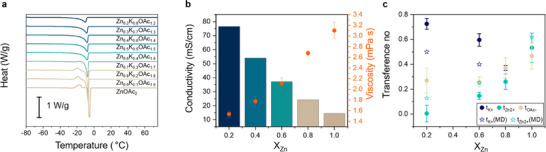
Physicochemical properties of the electrolytes Zn_X_K_1−X_(OAc)_1+X_ 30H_2_O, for 0<X_Zn_<1. a) DSC data collected from −80 to 80 °C (phase transitions T_g_ is a positive peak, and T_m_ is a negative peak). b) Viscosity and conductivity changes as a function of Zn fraction (X_Zn_). c) Transference number values as obtained from the Hittorf‐ICP‐OES technique for electrolytes with different Zn fraction (circles), and MD‐extracted transference numbers for zinc and potassium (stars).

First, we studied thermal properties of the electrolytes: Differential Scanning Calorimetry (DSC) profiles can be seen in **Figure** **1**a. For Zn‐rich systems, an exothermic peak at ≈−16 °C is observed before the melting point. This peak is often correlated to the crystal nucleation and growth. The decrease of this peak with an increase in K^+^ reflects a phase transition to a more disordered single‐phase frozen brine at lower Zn fractions.^[^ ^38^, ^39^ ^]^ The melting temperature (T_m_) increases from −5.5 °C (X_Zn_ = 1) to −9.9 °C (X_Zn_ = 0.2, Figure S1, Supporting Information) as Zn fraction decreases which can be attributed to changes in the ionic interactions,^[^ ^40^, ^41^ ^]^ solvation structure,^[^ ^39^ ^]^ and overall system disorder^[^ ^42^ ^]^ as the composition shifts from Zn‐rich to K‐rich system.

As shown in Figure 1b and Table S1 (Supporting Information), the electrolyte's viscosity decreases by a factor of 2 as the X_Zn_ fraction decreases, dropping from ≈3 mPas (X_Zn_ = 1) to ≈1.5 mPas (X_Zn_ = 0.2). This trend aligns with the DSC data, suggesting weaker inter‐ionic interactions and increased disorder in the electrolyte structure at lower Zn content. Concurrently, the conductivity increases sharply, from 14.4 mS cm^−1^ (X_Zn_ = 1) to 76.5 mS cm^−1^ (X_Zn_ = 0.2) which is still notably below the value of 138 mS cm^−1^ for the pure KOAc·30H_2_O solution (measured at 25 °C). At first glance, the high conductivity, increased disorder, and lower viscosity of potassium‐rich electrolytes might suggest superior transport properties for battery applications—but a closer examination of Zn‐specific transport properties reveals a more complex picture (discussed below).

### 2.2 Zn^2+^ Transport Properties and Solvation Environment

Next, we evaluated Zn‐specific transport by measuring its transference number (t_Zn_) in our electrolyte series at varying Zn:K ratios. As discussed, to address the challenges associated with measuring transference numbers in multicomponent electrolytes with dynamic interfaces, we developed a robust approach based on a modified Hittorf method. This method, detailed in the Experimental Section, utilizes the Hittorf cell (Figure S2, Supporting Information)^[^ ^14^, ^17^, ^43^ ^]^ and employs inductively coupled plasma ‐ optical emission spectroscopy (ICP‐OES) to quantify changes in the Zn and K concentrations in each compartment due to electromigration. By analyzing these concentration changes, we can accurately determine transference numbers not only for Zn^2+^ but also for the supporting cation (K^+^) and anion in the electrolyte.

Figure 1c and Table S2 (Supporting Information) show a rapid decrease in the Zn^2+^ transference number (t_Zn_) as the KOAc fraction increases. In pure Zn(OAc)_2_ (X_Zn_ = 1), the t_Zn_ = 0.533 ± 0.117, which is in good agreement with the value for Zn(OAc)_2_ under infinite dilution.^[^ ^44^ ^]^ Introducing K^+^ at a 0.2 fraction (X_Zn_ = 0.8) causes t_Zn_ to drop significantly to 0.260 ± 0.062, while the K^+^ transference number (t_K_) is 0.370 ± 0.021. At X_Zn_ = 0.2, the t_Zn_ decreases down to 0.005 ± 0.067, while t_K_ rises to 0.725 ± 0.043 and t_OAc_ = 0.270 ± 0.101, indicating that Zn‐ionic species are transported primarily by diffusion in this electrolyte. These results highlight a key trend: while electrolytes with high K fractions have higher total conductivity (Figure 1b), Zn^2+^ contributions to total ion transport are substantially reduced in those. Thus, Zn‐specific ionic conductivity, which is a product of total conductivity and Zn^2+^ transference number remains relatively high only at Zn fractions of X_Zn_≥0.6 (Figure S3, Supporting Information).

**Figure 2:**
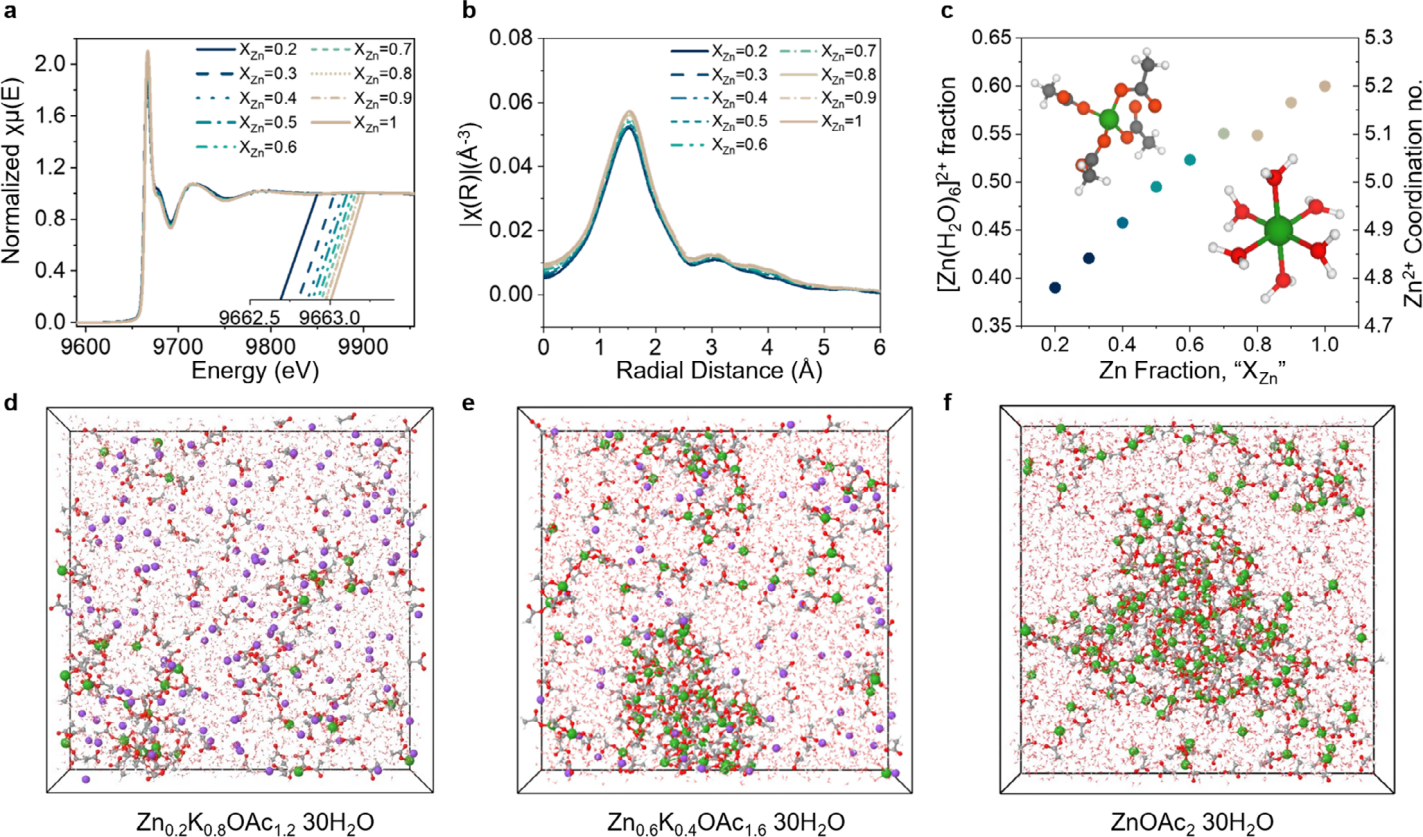
Zn^2+^ coordination environment studied by X‐ray absorption spectroscopy (XAS) as a function of electrolyte composition Zn_X_K_1‐X_(OAc)_1+X_ 30H_2_O, where 0<X<1. a) Normalized XAS plot and an inset including a zoom‐in of the XAS region. b) EXAFS changes in radial space. c) Zn^2+^ coordination environment and Zn^2+^ coordination numbers obtained from EXAFS fitting following.^[^ ^8^ ^]^ d–f) MD simulation boxes for the electrolytes with X_Zn_ = 0.2, 0.6, and 1 (Color scheme: green – Zn, violet – K, red – O, grey – C).

To better understand the observed Zn transference number trends and their connection to electrolyte structure, we examined how the Zn solvation environment changes with electrolyte composition using X‐ray absorption spectroscopy (XAS). **Figures** **2**a and S4a (Supporting Information) show that as the KOAc fraction increases, the Zn K‐edge shifts by ≈0.4 eV to lower energy (see Figure 2a inset), indicating a more electronegative Zn^2+^ coordination environment due to the partial displacement of the H_2_O molecules by the acetate anions (on average).^[^ ^8^ ^]^

Extended X‐ray absorption fine structure (EXAFS) analysis (Figure 2b) confirms this observation, showing a decrease in the Zn‐O peak intensity at ≈1.5 Å with increasing KOAc content. To quantify these changes, we fit the coordination environments at X_Zn_ = 0.2 and X_Zn_ = 1 with a pre‐optimized model^[^ ^8^ ^]^ comprising (1‐y)Zn(OAc)_4_^2−^ + yZn(H_2_O)_6_^2+^ (Figure S5, Table S3, Supporting Information), where “y” is the fraction of Zn^2+^ coordinated by water (Zn(H_2_O)_6_^2+^). The presence of isosbestic points in the EXAFS data (Figure S4, Supporting Information) allowed for linear combination fitting for intermediate compositions (Figure S6, Table S4, Supporting Information).^[^ ^8^ ^]^ As shown in Figure 2b, the decreasing Zn‐O intensity corresponds to a reduction in the coordination number (CN): the transition from water‐rich to acetate‐rich Zn^2+^ solvation is reflected in the CN change from 5.2 at X_Zn_ = 1 (y = 0.6) to 4.8 at X_Zn_ = 0.2 (y = 0.4), see Figure 2c, providing strong evidence of increased ion‐pair formation at higher KOAc fractions. These findings are in agreement with the relatively high donor number of acetate anions, compared to other non‐corrosive anions, and resulting favored formation of contact ion pairs.^[^ ^45^ ^]^ Notably, as KOAc concentration increases, the evolving solvation environment of Zn^2+^ mirrors the changes in t_Zn_ (Figure 1c). The decrease in Zn(H_2_O)_6_^2+^ species and the corresponding increase in ion‐paired Zn(OAc)_4_^2−^ complexes result in less mobile Zn^2+^, thus contributing to the Zn transference number reduction.

Molecular dynamics (MD) simulations provided additional insight into the structure and transport of electrolytes for X_Zn_ = 0.2, 0.6, and 1.0 at 333 K that was chosen to speed up equilibration. Snapshots of MD simulation boxes are shown in Figure 2d–f, while the most representative clusters are shown in Figure S7 (Supporting Information). A wide distribution of Zn^2+^ solvates is observed, with Zn(H_2_O)_6_^2+^, Zn(OAc)(H_2_O)_5_^+^, Zn(OAc)_2_(H_2_O)_4_ solvates having near octahedral coordination (Zn‐O CN = 6), while solvates with 3 or 4 OAc^−^ bound to zinc showing a near tetrahedral geometry (Zn‐O CN = 4). A fraction of the octahedrally coordinated Zn^2+^ is close to 30–50% in MD simulations with the other part of solvates being tetrahedrally coordinated. It is in good agreement with the analysis of EXAFS data that yielded fractions of the octahedrally and tetrahedrally coordinated Zn^2+^ solvates being in the range of 40–60%, albeit using a simpler (1‐y)Zn(OAc)_4_^2−^ + yZn(H_2_O)_6_^2+^ model. MD simulations yielded conductivities 19–41% higher than experiments as shown in Table S5 (Supporting Information). We consider this to be a sufficiently good prediction. It is worth noting that the transference number approximated from self‐diffusion coefficients (see Table S5, Supporting Information) assumes uncorrelated ionic motion without ion pairing or aggregation, thus systematically overestimating the experimentally measured transference number as ion‐pairs and larger ionic clusters are observed in both MD simulations and EXAFS experiments.  Nevertheless, the transference numbers from MD‐based self‐diffusion coefficients closely follow the trends observed experimentally, showing a significant decrease of t_Zn_ as the potassium fraction increases.

### 2.3 Zn Deposition Morphology

**Figure 3:**
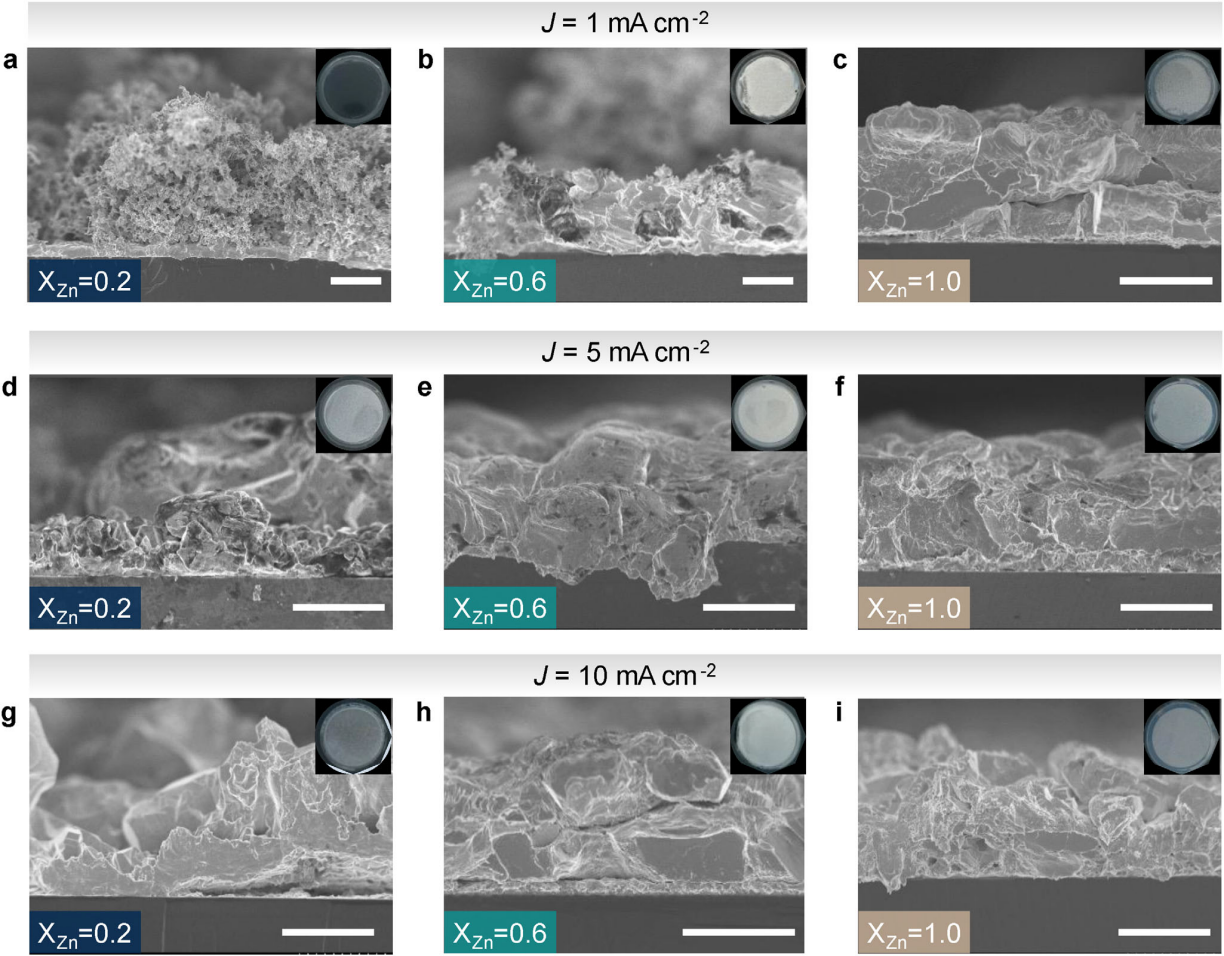
Transversal SEM visualization for the plating of 5 mAh cm^−2^ on Si/Zn substrates (200 nm Zn) at different current densities for the electrolytes Zn_X_K_1‐X_OAc_1+X_ ·30H_2_O, for 0.2<X_Zn_<1. Scale bar corresponds to 10 µm. a‐c) Deposition current density of 1 mA cm^−2^. d‐f) Deposition current of 5 mA cm^−2^. g‐i) Deposition current density of 10 mA cm^−2^. Insets show digital photographs of the deposits from the top.

To correlate the transference number with the Zn deposition morphology, we compared Zn deposits obtained after single plating in electrolytes with different Zn fraction (X_Zn_ = 0.2, 0.6, 1) under current densities of 1, 5, and 10 mA cm^−2^ with a plating capacity of 5 mAh cm^−2^ (Figure S8, Supporting Information). At 1 mA cm^−2^_,_ the density and homogeneity of the Zn deposits differ markedly, as seen from transverse (**Figure** **3**a–c) and top‐view (Figure S9, Supporting Information) scanning electron microscopy (SEM) images. In Zn(OAc)_2_·30H_2_O (X_Zn_ = 1) conformal deposition with ≈60% density is achieved (Figure 3c; Figure S10c, Supporting Information). In contrast, in Zn_0.2_K_0.8_(OAc)_1.2_·30H_2_O electrolyte, highly porous (“mossy”) Zn deposition with a density ≈20% is observed (Figure S10a, Supporting Information). At intermediate composition Zn_0.6_K_0.4_(OAc)_1.6_·30H_2_O, the Zn deposition exhibited significant heterogeneity (Figure 3b; Figure S10b, Supporting Information), displaying both “mossy” and flatter regions, resulting in a lower deposit density of ≈30% (Figure S10b, Supporting Information).

At higher deposition rates of 5 and 10 mA cm^−2^, homogenous and compact Zn deposition with ≈67 and 85% density was maintained in Zn(OAc)_2_·30H_2_O (X_Zn_ = 1, Figure S10f,i, Supporting Information). In contrast, in electrolytes with low Zn fraction (X_Zn_ = 0.2) we observed heterogenous deposition: while overall the Zn deposits appeared more conformal and denser (Figure 3d,g; Figures S9d,g and S10d,g, Supporting Information) during deposition we observed significant detachment and visible suspension of the metallic Zn deposits within the electrolyte, indicative of localized formation of Zn dendrites. In Zn_0.6_K_0.4_(OAc)_1.6_·30H_2_O electrolyte, the improved deposition density (69% at 5 mA cm^−2^ and 80% at 10 mA cm^−2^, Figure S9, Supporting Information), and notably flatter morphology is observed. The observed flatter morphology at higher Zn deposition currents can be explained by reduced nucleation sites formation at higher current densities (higher overpotentials), resulting in larger crystallites and denser deposits.^[^ ^46^, ^47^ ^]^ Overall, we observe that higher t_Zn_ in electrolytes leads to the formation of more uniform and denser Zn deposits.

### 2.4 Zn Plating/Stripping Performance

**Figure 4:**
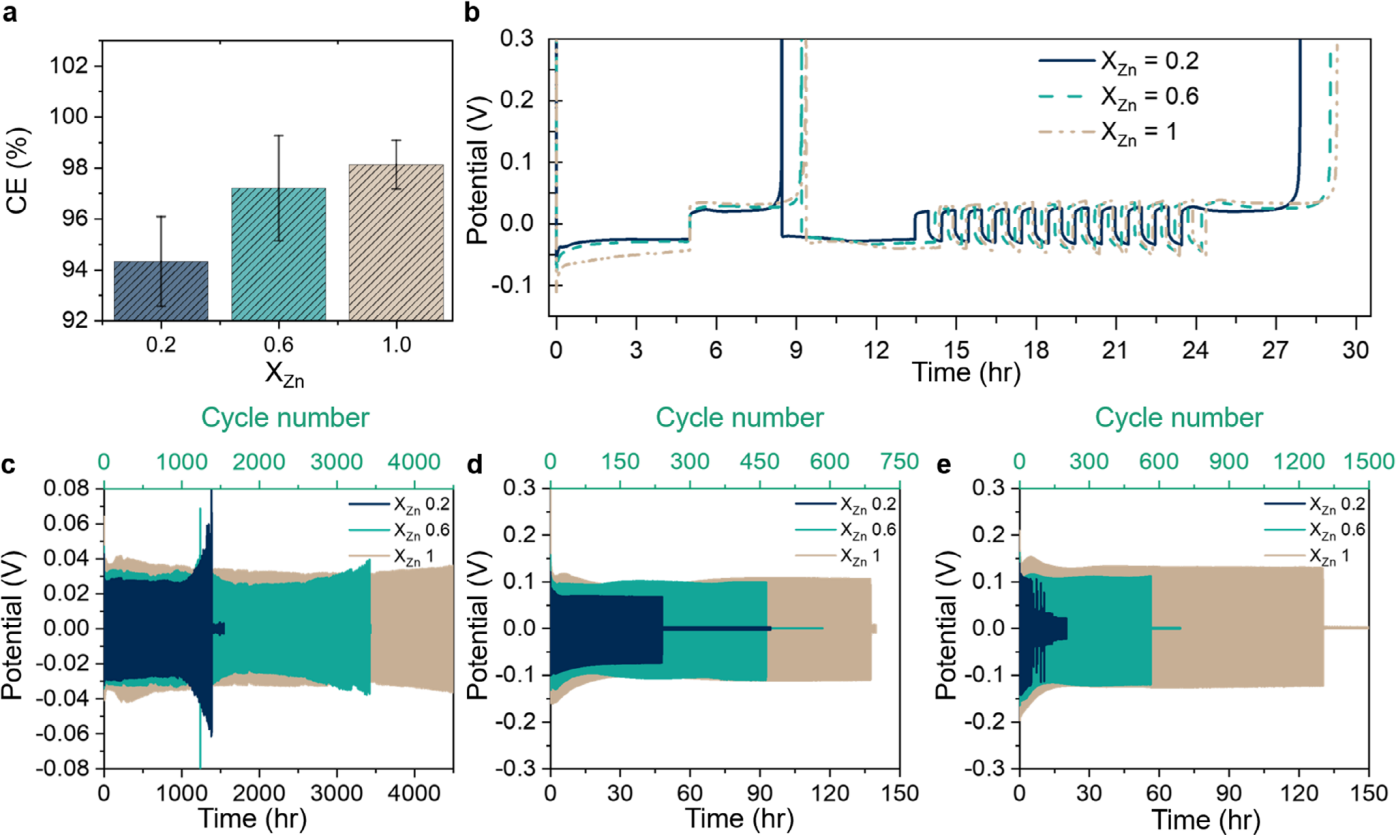
Electrochemical performance of Zn_X_K_1‐X_(OAc)_1+X_ ·30H_2_O, electrolytes across different compositions (0.2 < X_Zn_ < 1). a) Coulombic efficiency evaluated using the Aurbach protocol. Pre‐cycling at 1 mA cm^−2^ for 5 mAh cm^−2^, followed by cycling at 1 mA cm^−2^ for 0.5 mAh cm^−2^. b) Representative Aurbach profiles collected at 1 mA cm^−2^ for 0.5 mAh cm^−2^. c–e) Symmetric Zn||Zn cell cycling at current densities of 1, 5, and 10 mA cm^−2^, highlighting the impact of electrolyte composition on long‐term stability and plating/stripping behavior.

To assess the impact of Zn content on electrochemical performance, we evaluated Zn plating and stripping efficiencies using the Aurbach protocol in electrolytes with X_Zn_ = 0.2, 0.6, and 1. Coulombic Efficiency (CE) increases with increased Zn fraction and electrolyte t_Zn_ (**Figure** **4**a,b): 94.3% (X_Zn_ = 0.2), 97.8% (X_Zn_ = 0.6), and 98.1% (X_Zn_ = 1). The improvement in CE is more pronounced between X_Zn_ = 0.2 and X_Zn_ = 0.6, than in X_Zn_ = 0.6 and X_Zn_ = 1.0, even though the difference in transference number values is smaller in the former case than in the latter. This can be attributed to the similar Zn^2+^ specific conductivity in the electrolytes with X_Zn_≥0.6, but a significantly lower Zn^2+^ conductivity in the X_Zn_ = 0.2 electrolyte (Figure S3, Supporting Information). These findings are in agreement with recent studies on WiS electrolyte systems, where Zn^2+^ specific ionic conductivity was found to be the governing parameter for improved performance.^[^ ^33^ ^]^

Finally, to investigate the impact of Zn transference number (t_Zn_) on dendrite formation and cycling stability, we conducted long‐term symmetric Zn–Zn cycling tests at varying current densities (1, 5, and 10 mA cm^−2^) and rate capability tests at practical capacity (3 mAh cm^−2^). These current densities represent practical charging rates, including fast charging conditions,^[^ ^48^ ^]^ which significantly reduce the Sand's time (Figure S11, Supporting Information) and are expected to amplify the influence of t_Zn_ on concentration gradients and therefore dendrite formation.^[^ ^19^ ^]^ As shown in Figure 4c–e, electrolytes with higher t_Zn_ values exhibited significantly enhanced cycling stability across all current densities. At 1 mA cm^2^ and 0.5mAh cm^−2^, the cell with Zn_0.2_K_0.8_(OAc)_1.2_·30H_2_O electrolyte cycled for ≈1390 h (1400 cycles) before failure, while the cell with Zn_0.6_K_0.4_(OAc)_1.6_·30H_2_O cycled for ≈3150 h (3300 cycles), although a potential micro‐short circuit may have occurred ≈1240 h (see Zoom in Figure S12, Supporting Information). The Zn(OAc)_2_·30H_2_O electrolyte with X_Zn_ = 1 enabled the best cycling stability, operating for over 4500 h (4500 cycles) with no short‐circuit (still functioning at the time of writing). At higher current densities 5 mA cm^−2^ and 10 mA cm^−2^, the trends remain the same: the higher the t_Zn_ of the electrolyte, the longer the cell can cycle before short‐circuiting due to dendrite formation (see Figure S13, Supporting Information). However, the total number of cycles decreases drastically. The rate capability tests (see Figure S14, Supporting Information) showed early failure (at 10 mA cm^−2^) in the electrolyte with the lowest Zn fraction (X_Zn_ = 0.2, low t_Zn_). The cell with X_Zn_ = 0.6 electrolyte failed at 20 mA cm^−2^, while the X_Zn_ = 1 electrolyte facilitated completion of the full test protocol. These results showcase the importance of high t_Zn_ for practical Zn‐metal batteries.

## 3 Conclusion

Our study demonstrates that while adding KOAc markedly boosts overall ionic conductivity, it dramatically lowers the Zn^2+^ transference number — a trade‐off that negatively affects Zn metal anodes during cycling. Using a modified Hittorf method, we accurately quantify transference numbers in complex Zn‐K acetate electrolytes and, with the aid of X‐ray absorption spectroscopy and MD simulations, established how evolving Zn^2+^ solvation environments correlate with the reduced Zn^2+^ mobility. By systematically varying the Zn^2+^ fraction, we show that higher Zn^2+^ transference numbers and Zn‐specific ionic conductivity yield more uniform and dense Zn deposits, improved cycling stability, and minimized dendrite formation even at high current densities. These insights underscore the necessity of quantitatively measuring and optimizing Zn^2+^ transference, alongside with total conductivity, when designing aqueous Zn‐metal battery electrolytes. Moving forward, this framework provides a robust approach for guiding electrolyte formulations that balance conductivity gains with targeted Zn^2+^ transport properties, ultimately paving the way for safer, longer‐lived Zn‐based batteries.

## 4 Experimental Section

### Materials

High‐purity zinc foil (>99.95%) was acquired from Goodfellow, high‐purity zinc rods (⌀4.8 mm, >99.99%) were acquired from Merck. The TOPAS (cyclic polyolefin) microfluidic channels for XAS were purchased from ChipShop Germany. Zinc and potassium atomic absorption spectroscopy (AAS) standard solutions (Specpure 1000 µg mL^−1^) were purchased from ThermoFisher Scientific. Potassium acetate (>99%), zinc acetate dihydrate (>99%), and other chemicals were acquired from Sigma–Aldrich.

### Electrolyte Conductivity

The conductivity was measured using an Oakton 2700 benchtop conductivity meter.

### Viscosity

The measurements were carried out on an ARES‐G2 oscillatory rheometer from TA Instruments, using a cone‐plate geometry for samples with higher viscosity. Shear rates varied between 10 and 1000 Hz, with viscosity values determined from measurements at frequencies above 100 Hz.

### Differential Scanning Calorimetry (DSC)

The thermal properties of the electrolytes were evaluated using a DSC 2500 (TA Instruments). Samples were initially cycled once from −80 to 80 °C, followed by a second cycle back to −80 °C at a rate of 10 °C per minute. Data was subsequently collected from −80 to 80 °C at a slower rate of 1 °C per minute.

### X‐Ray Absorption Spectroscopy (XAS)

Following the same procedure as in our previous work,^[^ ^8^ ^]^ X‐ray absorption spectroscopy (XAS) measurements were conducted at the Swiss Light Source (SLS) at the Paul Scherrer Institute (PSI) on the X10DA‐SuperXAS beamline. Data sets were collected in Quick Scanning XAS mode around the Zn K‐edge, with Zn metal used for calibration during a 5‐min scan.^[^ ^49^ ^]^ The electrolyte was continuously pumped through a microfluidic channel at a flow rate of 100 µL min^−1^ to minimize beam‐induced damage. Data analysis was performed using the Demeter software package. X‐ray absorption near edge structure (XANES) normalization was achieved by setting the edge energy (E°) at the zero of the second derivative and manually adjusting the pre‐ and post‐edge regions. Extended X‐ray absorption fine structure (EXAFS) data were processed over a wave number range of 3–12 Å^−1^ to maintain a low signal‐to‐noise ratio, with results presented using a k^3^‐weighting. For fitting, the amplitude reduction factor (S_0_^2^) was set to 1, as established in prior studies.^[^ ^50^ ^]^

### Transference Number Measurements—Modified Hittorf Method

The transference number quantification was carried out using a modified Hittorf method with a custom‐built three‐compartment glass Hittorf cell (Figure S2, Supporting Information). The cell consisted of cathodic and anodic compartments connected to a U‐shaped middle chamber via two Teflon valves. A third Teflon valve located in the middle chamber was used for filling up and emptying.

### (a) Cell Assembly

To prevent intermixing of electrolyte from different compartments, the openings of the valves connecting the anodic/cathodic and middle chamber were tightly packed with filter paper (Whatman B2, ⌀ 4 mm) while ensuring sufficiently low cell resistance. The middle chamber was then filled with the electrolyte with the connecting valves in the open position, ensuring no air bubbles were trapped in the valve openings. A specified volume (2.3 mL) of the electrolyte was added to the anodic and cathodic chambers.

### (b) Electrode preparation

Zinc electrodes (⌀4.8 mm rods or strips) were polished, cleaned with ethanol, and masked with heat‐shrinking tubing (⌀ 6 mm) to expose a surface of 2.83 cm^2^ (≈7 mm of rod length exposed). The electrodes were pre‐conditioned in the desired electrolyte to avoid high surface reactivity during the experiment. Right before the start of an experiment, the electrodes were dried with Kim wipes and weighed. The electrodes were inserted through a cut septum and placed in the corresponding chamber.

### (c) Galvanostatic polarization

The assembled cells were placed in a temperature‐controlled chamber (Binder) at 24 °C. Galvanostatic polarization was applied using a potentiostat (Admiral Plus Squidstat or Biologic MPG‐2). The total charge passed was limited to 4–5% of the zinc ions in the anodic chamber to ensuring that a sufficient change in Zn concentration (>3%) takes place for the following ICP‐OES measurement, while minimizing the diffusion effects.

### (d) Sample collection

After the measurement was completed, the valves were closed to isolate the compartments, and the electrolyte from each chamber was collected in a separate vial for analysis. Each measurement was repeated at least three times.

### Concentration Analysis using Inductively Coupled Plasma Optical Emission Spectroscopy (ICP‐OES)

The concentration of the Zn^2+^ and K^+^ in the electrolytes from the Hittorf measurements were quantified using ICP‐OES (Horiba ULTIMA2 or Agilent Varian 720‐ES).

### (a) Sample preparation

Samples were prepared for analysis via two‐step dilution. The first dilution step was carried out immediately after the experiment, with a 10 vol% aqueous solution of nitric acid as the diluent to lower the pH of the sample and prevent precipitation of any salts. The second dilution was carried out using ultrapure water (18.2 MΩ·cm) to achieve a target concentration of 20–100 ppm (both for Zn and K).

### (b) Measurements

The instrument was calibrated using 0, 10, 25, 50, and 100 ppm calibration standards for both Zn^2+^ and K^+^ (prepared by diluting Zn^2+^ and K^+^ standard solutions from 1000 µg mL^−1^). The instrument was recalibrated between each set of measurements (10–12 samples) using the calibration standards mentioned above. All samples were measured three times.

### (c) Sample concentrations

Using the calibration curves for Zn and K, concentrations prior the dilution were back‐calculated for anodic, cathodic, and middle compartments. As the Hittorf method assumes negligible concentration changes in the middle chamber, values from this compartment were compared to the control (as‐prepared electrolyte), and measurements with a deviation > 2.5% between these two concentrations were excluded from further analysis.

### Calculations of transference numbers

Calculations were based on the change in the ion concentration in the anodic compartment to avoid potential inaccuracies due to concurrent hydrogen evolution that can take place in the cathodic chamber. The transference numbers were calculated based on the molar balance in the chamber. The molar balance for Zn^2+^ in the anodic chamber is expressed as:

(1) $$n_{Zn,A}=n_{Zn}^{0}+n_{Zn,redox}-n_{Zn,migrated}$$

where *n*_*Zn*,*A*_​ [mol] is *the* number of moles of Zn^2+^ in the anodic chamber after the experiment, $n_{Zn}^{0}$​ [mol] is *the* number of moles of Zn^2+^ in the anodic chamber before the experiment, *n_migration_​* [mol] is the number of moles transported via migration, *n*_*Zn*,*redox*_ [mol] is the number of moles generated due to electro‐dissolution in the anodic chamber.

By applying Faraday's law and the definition of transference number, the Zn^2+^ transference number (t_Zn_) can then be calculated as follows

(2) $$n_{Zn,A}=n_{Zn}^{0}+\frac{Q}{zF}-t_{Zn}\frac{Q}{zF}$$

(3) $$t_{Zn}=\frac{\left(n_{Zn}^{0}-n_{Zn,A}\right)\;\;2F}{Q}+1$$

(4) $$t_{Zn}=\frac{\left(c_{Zn}^{0}-c_{Zn,A}\right)v\;2F}{W_{Zn}Q}+1$$

where $c_{Zn}^{0}$ [g/L] is the concentration of Zn^2+^ in the anodic chamber before the experiment, which should be close to the concentration measured in the middle chamber after the experiment, *c*_*Zn*,*A*_ [g/L] is the concentration of Zn^2+^ in the anodic chamber after the experiment, *v* [L] is the volume of electrolyte in the anodic chamber, *Q* [C] is the total charge passed during the experiment, *z* is the number of electrons in the reaction (2 for Zn^2+^, 1 for K^+^),  *W_Zn_* =  65.38 g/mol is the molecular weight of Zn, *F* = 96485 C mol^−1^ is the Faraday constant.

Similarly, molar balance for K^+^ yields the following K^+^ transference number (t_K_) equation:

(5) $$n_{K,A}=n_{K}^{0}-n_{K,migrated}$$

(6) $$t_{K}=\frac{\left(n_{K}^{0}-n_{K,A}\right)F}{Q}$$

(7) $$t_{K}=\frac{\left(c_{K}^{0}-c_{K,A}\right)vF}{W_{K}Q}$$

Since the acetate anion cannot be detected via ICP‐OES, its transference number was inferred using the definition that the total sum of transference numbers equals 1:

(8) $$t_{OAc}=1-t_{K}-t_{Zn}$$

### Sand's Time

The Sand's time was estimated using the Sand's formula:^[^ ^19^, ^51^ ^]^

(9) $$t_{Sand}=\frac{\pi D_{app}{\left(z_{Zn}C_{0}F\right)}^{2}}{4{\left(J\left(1-t_{Zn}\right)\right)}^{2}}$$

Where *D_app_* is the apparent diffusion coefficient, *z_Zn_* is the charge number of Zn^2+^, *C*_0_ is the initial salt concentration at the bulk, *F* is Faraday's constant, *J* is current density and *t_Zn_* is the cation transference number. To perform the calculations, MD extracted diffusion coefficients were employed.

### Aurbach Protocol

Cells were assembled in a two‐electrode PFA Swagelok‐like configuration using copper (Cu) rods as current collectors and 0.5‐inch electrodes with separators. A compressed Whatman glass fiber separator (0.5 mm thick) separated the working and counter electrodes. The working electrode was a 5 µm‐thick high‐purity Cu foil, and the counter electrode was a 250 µm‐thick Zn foil. Each cell contained 150 µL of electrolyte.

Cells were cycled according to the Aurbach protocol. First, 5 mAh cm^−^^2^ of Zn was plated and stripped at a current density of 1 mA cm^−2^. Next, a Zn reservoir of 5 mAh cm^−2^ was plated, followed by 10 plating/stripping cycles at 1 mA cm^−2^ with a charge of 0.5 mAh cm^−2^ per cycle. Finally, a stripping step was performed at 1 mA cm^−2^ with a cut‐off charge of 5 mAh cm^−2^.

### Symmetric Zn–Zn Cycling and Rate Capability Tests

Symmetric cells were assembled in 0.25‐inch Swagelok cells using copper (Cu) current collectors and a Whatman glass fiber separator. Each cell contained 30 µL of electrolyte, and the Zn disk electrodes with 0.25‐inch diameter and a thickness of 250 µm. The cells were cycled at current densities of 1, 5, and 10 mA cm^−2^, with 0.5 mAh cm^−2^ charge for each cycle. Rate capability tests were carried out in the same cell assemblies as in the symmetric Zn–Zn cycling. The cells were cycled at 3 mAh cm^−2^ charge for each cycle with the following procedure: first five cycles at 1 mA cm^−2^, ten cycles each at 2, 5, 10, and 20 mA cm^−2^, then cycling at 1 mA cm^−2^ until failure.

### Scanning Electron Microscopy (SEM) Studies: Cross‐Section Visualization

To visualize the cross‐sectional Zn deposition morphology, Zn deposits were prepared on Si wafers with 200 nm of Zn metal evaporated. These Si wafers with 200 nm Zn served as working electrodes in a custom PEEK cell with a cylindrical opening (1.5 cm diameter × 1 cm height) and a 250 µm thick Zn foil served as the counter electrode. Next, Zn was electrodeposited at current densities of 1, 5, and 10 mA cm^−2^ and a total charge of 5 mAh cm^−2^. After Zn electrodeposition, the samples were fractioned from the back side of the Si wafer using a diamond cutter, ensuring that the electrodeposited Zn remains unaltered. SEM images were collected using a Hitachi S‐4800 SEM microscope to assess the morphology.

The deposition density was estimated from the average deposition height in the cross‐sectional SEM images (Figure S10, Supporting Information). Since the deposited charge is 5 mAh cm^−2^ and the density of Zn metal is 7.14 g cm^−2^, yielding the resulting deposit height of ≈8.4 µm for fully dense Zn. However, due to measurement uncertainties stemming from the localized nature of this method, it primarily serves as a comparative tool for evaluating difference in Zn deposits obtained in different electrolytes.

### Molecular Dynamics Simulations (MD)

Molecular dynamics simulations were performed with Tinker9 (git commit 70bd052) using the AMOEBA09 polarizable force field.^[^ ^52^ ^]^ The default mixed‐precision executable was compiled with CUDA/11.6 on Nvidia A100 GPUs. Force field parameters were refined further from a previous publication^[^ ^8^ ^]^ to reduce the mean unsigned error in interaction energies computed across 18 clusters, sampling potassium‐water, zinc‐water, acetate‐water, potassium‐acetate, zinc‐acetate, potassium‐water‐acetate, and zinc‐water‐acetate clusters. Clusters with cation‐acetate interactions feature a mix of mono‐ and bidentate complexes. After further testing, the Rmin_ij for zinc – carboxylate oxygen interactions was tuned to predict a 1.5 kcal mol^−1^ stronger interaction energy for the [Zn(OAc)_4_]^2−^ complex as the force field parameters derived purely from a fit to ab initio data were too dissociating and predicted large errors in the conductivity. Compositions simulated comprised Zn_x_K_y_OAc_z_‐30H_2_O, where x = {0.2, 0.6, 1.0}, y = 1‐x, and z is adjusted for charge neutrality; four independent replicas were generated for each composition for better statistics. The number of waters was fixed for each composition (4800) and the number of ions were adjusted accordingly. Box sizes were equilibrated in the NPT ensemble using the Nose‐Hoover barostat and thermostat with default coupling constants and a 1 fs timestep at 333.15 Kelvin and 1 atmosphere pressure. Densities were considered converged when the 4 independent replicate runs for each concentration showed no drift in average box size versus time. This occurred only for the box for the Zn_x_K_y_OAc_z_‐30H_2_O, where x = 0.2 compositions after 150 nanoseconds – average box size over the last 50 nanoseconds across 4 independent replicates agreed to 2‐decimal places. After equilibrating, all 4 replicates of the x = 0.2 composition were scaled to the same box size with simulations performed for 1000 additional nanoseconds using the RESPA multi time‐step integrator, Bussi thermostat (333.15 Kelvin) and 2 femtosecond timestep. The compositions with higher zinc content Zn_x_K_y_OAc_z_‐30H_2_O, where x = 0.6, 1.0, oscillated between two slightly different density packing states and so all properties are computed by continuing these trajectories in the NPT ensemble for 650 nanoseconds. Final analysis used up to 450‐600 nanoseconds of these trajectories with coordinates saved every 10 picoseconds (all compositions). The Ewald and vdW cutoffs were set to 10 Å with a PME‐grid mesh of 64^3^. The long range vdW correction was added and center of mass motion is removed at each timestep. Force field parameters and restart files (.xyz,.dyn) are provided in the Supporting Information.

## Conflict of Interest

The authors declare no conflict of interest.

## Supporting information



Supporting Information

Supporting Information

## Data Availability

The data that support the findings of this study are available from the corresponding author upon reasonable request.
