# Supplementary material for: Measuring Zn Transference with Precision: Insights for Dendrite‐Free Zinc Metal Anodes
Source: Adv Mater. 2025 Aug 23;37(46):e02245. doi: 10.1002/adma.202502245 (PMC12631524; doi:10.1002/adma.202502245)
Supplement: Supplementary file 1 — Supporting Information [file ADMA-37-e02245-s001.docx]

**SUPPLEMENTARY INFORMATION**

**Measuring Zn Transference with Precision: Insights for Dendrite-Free Zinc Metal Anodes**

Dario Gomez Vazquez^1†^, Julita Tabor^1†^, Travis P. Pollard^2^, Oleg Borodin^2^, Maria R. Lukatskaya^1, *^

^1^ Department of Mechanical and Process Engineering, ETH Zurich, 8092 Zurich, Switzerland

^2^ DEVCOM Army Research Laboratory, Battery Science Branch, Energy Sciences Division, Adelphi, MD, 20783, USA

*Correspondence: [mlukatskaya@ethz.ch](mailto:mlukatskaya@ethz.ch)

†These authors contributed equally to this work.

TABLE OF CONTENTS

[**Table S1**. Physicochemical properties 3](#_Toc204326913)

[**Table S2.** Transference number 3](#_Toc204326914)

[**Table S3.** EXAFS Fitting parameters for electrolytes with X_Zn_= 0.2 and 1. 3](#_Toc204326915)

[**Table S4.** Linear combination fitting (LCF) for the EXAFS model in X space. 4](#_Toc204326916)

[**Table S5.** MD-extracted transport properties 4](#_Toc204326917)

[**Table S6.** Sand’s time as function of electrolyte composition and deposition current density 4](#_Toc204326918)

[SUPPLEMENTARY DISCUSSION 5](#_Toc204326919)

[SUPPLEMENTARY FIGURES 6](#_Toc204326920)

[**Figure S1.** Melting temperature (Tm) as function of electrolyte composition for Zn_X_K_1-X_OAc_1+X_ 30H_2_O, with 0.2 ≤ X ≤ 1. 6](#_Toc204326921)

[**Figure S2.** Schematic of the Hittorf cell used for transference number measurements. 6](#_Toc204326922)

[**Figure S3.** Individual ion conductivities as extracted from the transference number as function of electrolyte composition, for Zn_X_K_1-X_OAc_1+X_ 30H_2_O, with 0.2 ≤ X ≤ 1. 7](#_Toc204326923)

[**Figure S4.** XAS (a) and EXAFS in χ space (b) showing isosbestic points for the electrolytes Zn_X_K_1-X_OAc_1+X_ 30H_2_O X=0.2-1. 7](#_Toc204326924)

[**Figure S5.** Fitting to Zn(OAc)_4_ and Zn(H_2_O)_6_ clusters for the electrolyte a-c) X_Zn_=0.2 and d-f) X_Zn_=1. 8](#_Toc204326925)

[**Figure S6.** Linear combination fitting for XAS as function of electrolyte composition a) fitting to X_Zn_=0.2 b) fitting to X_Zn_=1. 8](#_Toc204326926)

[**Figure S7.** Representative Zn solvates from MD simulations for X_Zn_ = 1.0 (a-f) and X_Zn_ = 0.2 (g-k). 9](#_Toc204326927)

[**Figure S8.** Plating profiles for the SEM visualizations as function of electrolyte composition Zn_X_K_1-X_OAc_1+X_ ·30H_2_O X_Zn_=0.2, 0.6 and 1. a) results at 1 mA/cm^2^ b) results at 5 mA/cm^2^ c) results at 10 mA/cm^2^. 10](#_Toc204326928)

[**Figure S9.** Top-view SEM visualization for the plating of 5 mAh/cm^2^ on Si/Zn substrates (200nm Zn) at different current densities for the electrolytes Zn_X_K_1-X_OAc_1+X_ 30H_2_O, for 0.2<X<1. **a-c)** Deposition current of 1 mA/cm^2^. **d-f)** Deposition current of 5 mA/cm^2^. **g-i)** Deposition current of 10 mA/cm^2^. The columns correspond to the electrolyte composition. 10](#_Toc204326929)

[**Figure S10.** Transversal SEM visualization with Zn plating density quantification for the plating of 5 mAh/cm^2^ (8.4 µm) on Si/Zn substrates (200 nm Zn) at different current densities for the electrolytes Zn_X_K_1-X_OAc_1+X_ 30H_2_O, for 0.2<X<1. **a-c)** Deposition current of 1 mA/cm^2^. **d-f)** Deposition current of 5 mA/cm^2^. **g-i)** Deposition current of 10 mA/cm^2^. The columns correspond to the electrolyte composition. 11](#_Toc204326930)

[**Figure S11.** Sand’s time as function of deposition current and electrolyte composition for the electrolytes Zn_X_K_1-X_OAc_1+X_ 30H_2_O, with X=0.2, 0.6 and 1. 12](#_Toc204326931)

[**Figure S12.** Symmetric cycling zoom in depicting the first cycles and the moment of short-circuit for each cell as function of electrolyte composition. a-c. Show the cycling at currents of 1, 5 and 10 mA/cm^2^ for a charge of 0.5 mAh/cm^2^ respectively. 12](#_Toc204326932)

[**Figure S13.** Symmetric cycling duplicates depicting reproducibility and cell to cell variation. a-c. Show the cycling at currents of 1, 5 and 10 mA/cm^2^ for a charge of 0.5 mAh/cm^2^ respectively. 13](#_Toc204326933)

[**Figure S14.** Rate capability tests for electrolytes with X_Zn_ = 0.2, 0.6, 1. The cells were cycled to the capacity of 3 mAh/cm^2^ at current densities between 1 and 20 mA/cm^2^. 13](#_Toc204326934)

[**Figure S15.** Repetitions of the rate capability tests for electrolytes with X_Zn_ = 0.2 (n=3), 0.6 (n=4), 1 (n=4), where n is the number of test repetitions. The cells were cycled to the capacity of 3 mAh/cm^2^ at current densities between 1 and 20 mA/cm^2^. 14](#_Toc204326935)

**SUPPLEMENTARY TABLES**

## **Table S1**. Physicochemical properties

|  | Conductivity 25 °C | std. dev. | Conductivity 60 °C | std. dev. | Density | std dev | Viscosity | std. dev. |
| --- | --- | --- | --- | --- | --- | --- | --- | --- |
| X_Zn_ | mS/cm | mS/cm | mS/cm | mS/cm | g/cm^3^ | g/cm^3^ | mPa s | mPa s |
| 0 | 138 |  |  |  |  |  |  |  |
| 0.2 | 76.55 | 0.005 | 110.47 | 0.047 | 1.09 | 0.001 | 1.53 | 0.062 |
| 0.4 | 54.12 | 0.012 | 88.61 | 0.029 | 1.11 | 0.004 | 1.77 | 0.097 |
| 0.6 | 37.15 | 0.005 | 60.97 | 0.021 | 1.13 | 0.000 | 2.10 | 0.104 |
| 0.8 | 24.32 | 0.000 | 38.68 | 0.025 | 1.15 | 0.001 | 2.67 | 0.043 |
| 1 | 14.43 | 0.000 | 19.85 | 0.041 | 1.16 | 0.001 | 3.10 | 0.151 |

## **Table S2.** Transference number

|  | Transference number | | | | | |
| --- | --- | --- | --- | --- | --- | --- |
| X_Zn_ | t_K+_ | std. dev. | t_Zn2+_ | std. dev. | t_OAc-_ | std. dev. |
| 0.2 | 0.725 | 0.043 | 0.005 | 0.067 | 0.270 | 0.101 |
| 0.6 | 0.596 | 0.050 | 0.147 | 0.028 | 0.258 | 0.040 |
| 0.8 | 0.370 | 0.021 | 0.260 | 0.062 | 0.370 | 0.082 |
| 1 |  |  | 0.533 | 0.117 | 0.467 | 0.104 |

## **Table S3.** EXAFS Fitting parameters for electrolytes with X_Zn_= 0.2 and 1.

| X _Zn_ | DFT Model | Scattering path | Fraction “y” | error on y | CN | R (Å) Abs | error on R | s^2^ (Å^2^) | error on s^2^ | ∆E | error on DE | R value | R range | k range |
| --- | --- | --- | --- | --- | --- | --- | --- | --- | --- | --- | --- | --- | --- | --- |
| 0.2 | Zn(H_2_O)_6_ | Zn-Ow | 0.391 | 0.160 | 6 | 2.0804 | - | 0.0095 | 0.0029 | 4.74 | 1.6 | 0.02 | 1.3-3 | 3 to 13 |
|  | Zn(OAc)_4_ - mono | Zn-OAc | 0.609 | 0.160 | 4 | 1.9785 | - | 0.0059 | 0.0011 |  |  |  |  |  |
| 1 | Zn(H_2_O)_6_ | Zn-Ow | 0.602 | 0.156 | 6 | 2.0804 |  | 0.0099 | 0.0022 | 4.59 | 1.35 | 0.02 | 1.3-3 | 3 to 13 |
|  | Zn(OAc)_4_ - mono | Zn-OAc | 0.398 | 0.156 | 4 | 1.9785 |  | 0.0060 | 0.0016 |  |  |  |  |  |

## **Table S4.** Linear combination fitting (LCF) for the EXAFS model in X space.

|  | weight X_Zn_=0.2 | | weight X_Zn_=1 | |
| --- | --- | --- | --- | --- |
| X_Zn_ | LCF | std. dev. | LCF | std.dev. |
| 0.2 | 1 | 0 | 0 | 0 |
| 0.3 | 0.75 | 0.03 | 0.25 | 0.03 |
| 0.4 | 0.61 | 0.02 | 0.39 | 0.02 |
| 0.5 | 0.42 | 0.02 | 0.58 | 0.02 |
| 0.6 | 0.33 | 0.01 | 0.67 | 0.01 |
| 0.7 | 0.22 | 0.01 | 0.78 | 0.01 |
| 0.8 | 0.23 | 0.01 | 0.77 | 0.01 |
| 0.9 | 0.08 | 0.01 | 0.92 | 0.01 |
| 1 | 0 | 0 | 1 | 0 |

## **Table S5.** MD-extracted transport properties

|  | Transference number | | | Conductivity 60°C | [Zn(H_2_O)_6_]^2+^ fraction | Zn^2+^ coordination number |
| --- | --- | --- | --- | --- | --- | --- |
| X_Zn_ | t_K+_ | t_Zn2+_ | t_OAc-_ | mS/cm |  |  |
| 0.2 | 0.50 | 0.13 | 0.37 | 156.1 | 0.33 | 4.60 |
| 0.6 | 0.40 | 0.25 | 0.35 | 83.9 | 0.28 | 4.52 |
| 1 |  | 0.62 | 0.38 | 23.7 | 0.48 | 4.93 |

## **Table S6.** Sand’s time as function of electrolyte composition and deposition current density

|  | Sand’s time | | |
| --- | --- | --- | --- |
| J | s | | |
| mA/cm^2^ | X_Zn_= 0.2 | X_Zn_= 0.6 | X_Zn_= 1 |
| 1 | 24853 | 240437 | 833315 |
| 5 | 994 | 9617 | 33333 |
| 10 | 249 | 2404 | 8333 |

# SUPPLEMENTARY DISCUSSION

**Transference number measurements: Practical Considerations**

Obtaining accurate transference numbers requires careful attention to several key experimental details. The following guidelines cover critical aspects of experimental design and data validation.

- The Zn electrodes in anode and cathode compartments should have nearly identical surface area. The electrodes should have a smooth geometry, that can cause localized current hotspots and promote side reactions.
- The electrode area exposed to the electrolyte should be located below the connecting channel leading to the middle compartment. This can be achieved by enclosing the electrode in an insulating sleeve, such as heat-shrinking tubing.
- The intermixing of electrolyte between the cell compartments should be strictly avoided, even minor electrolyte transfer due to tilting of the cell or bubble release from the valves can significantly affect the results. For this reason, the openings of the valves between compartments were tightly packed with Whatman filter paper (~100 layers).
- When filling the middle chamber, electrolyte droplets may remain on the walls of the outlet channel. To prevent salt residues and their subsequent dissolution during emptying, the channel must be thoroughly rinsed with DI water and dried before starting the experiment
- The charge passed during the experiment should correspond to 4-5% of the Zn^2+^ concentration in one of the chambers. Lower amounts of charge would induce concentration changes not large enough to be distinguishable by ICP-OES. Larger amounts of charge would induce diffusion impacts on the concentration.
- The current density should be low enough to minimize side reactions, but not so low as not excessively extend the experiment duration (to avoid ion diffusion between compartments). In this study, current densities between 0.4 and 0.6 mA were used, resulting in experiment times of 4-16 hours, depending on the electrolyte formulation.
- To ensure that concentration gradient not extending into the middle chamber, control ICP-OES measurements must be performed. Control samples for ICP-OES were prepared using as-prepared electrolyte, following the same dilution steps as for experimental samples (from Hittorf measurement), but without exposure to the cell.

The following points must be taken into consideration, when evaluating the results:

- Experimental runs should be disregarded if any volume changes take place in the chambers due to leakages or significant bubble formation.
- The middle and control chamber concentration should be nearly identical. A deviation of middle concentration forms the control concentration may indicate significant diffusion between compartments yielding incorrect transference number results. Therefore, we only utilized the experimental results where the deviation between the middle and control chambers was below 2.5%.

# SUPPLEMENTARY FIGURES


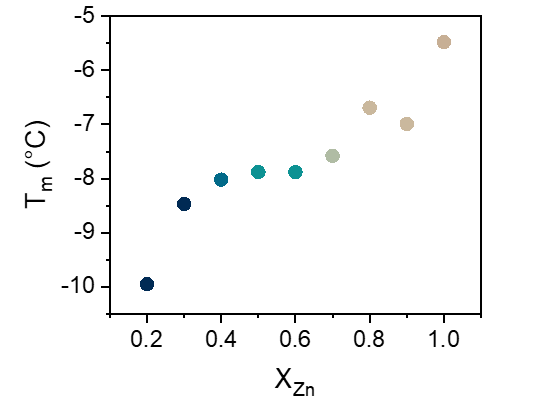


## **Figure S1.** Melting temperature (Tm) as function of electrolyte composition for Zn_X_K_1-X_(OAc)_1+X_ 30H_2_O, with 0.2 ≤ X ≤ 1.


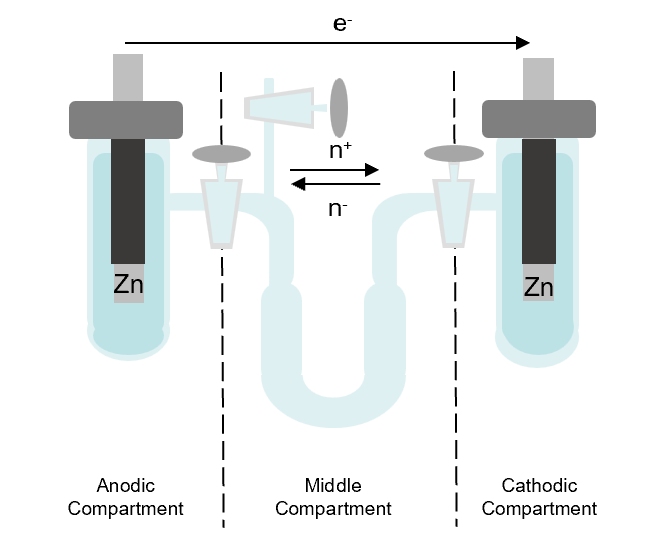


## **Figure S2.** Schematic of the Hittorf cell used for transference number measurements.

## **Figure S3.** Individual ion conductivities as extracted from the transference number as function of electrolyte composition, for Zn_X_K_1-X_(OAc)_1+X_ ·30H_2_O, with 0.2 ≤ X ≤ 1.


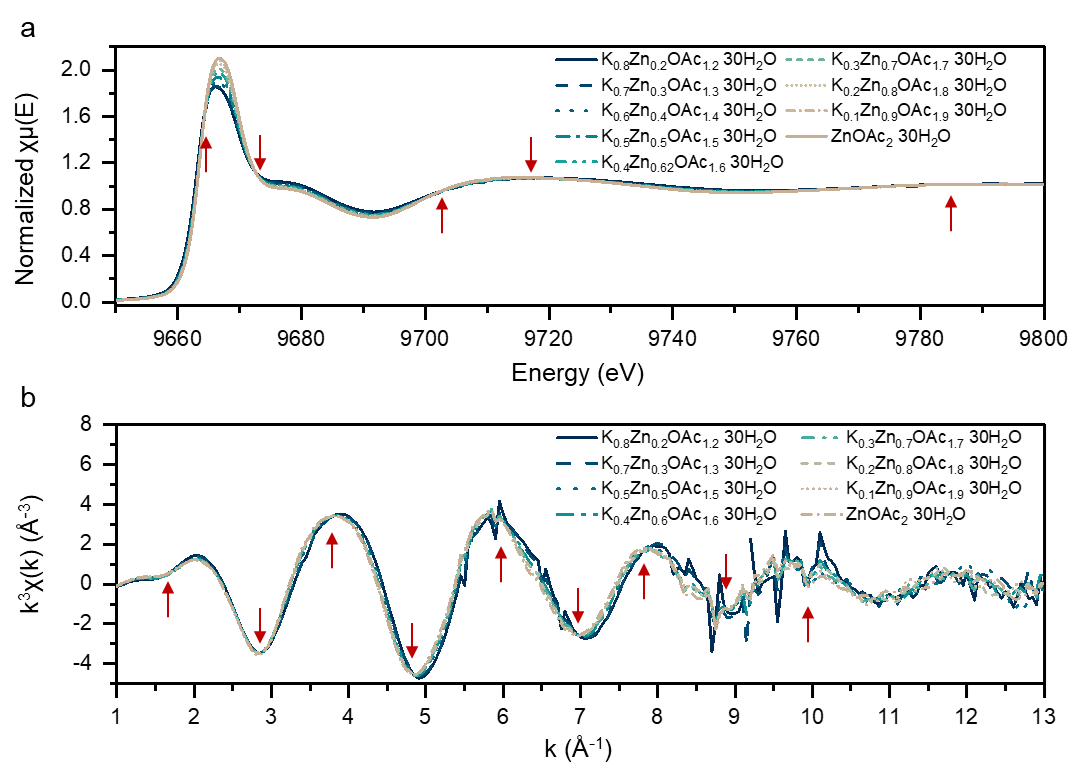


## **Figure S4.** XAS (a) and EXAFS in χ space (b) showing isosbestic points for the electrolytes Zn_X_K_1-X_(OAc)_1+X_ ·30H_2_O X=0.2-1.


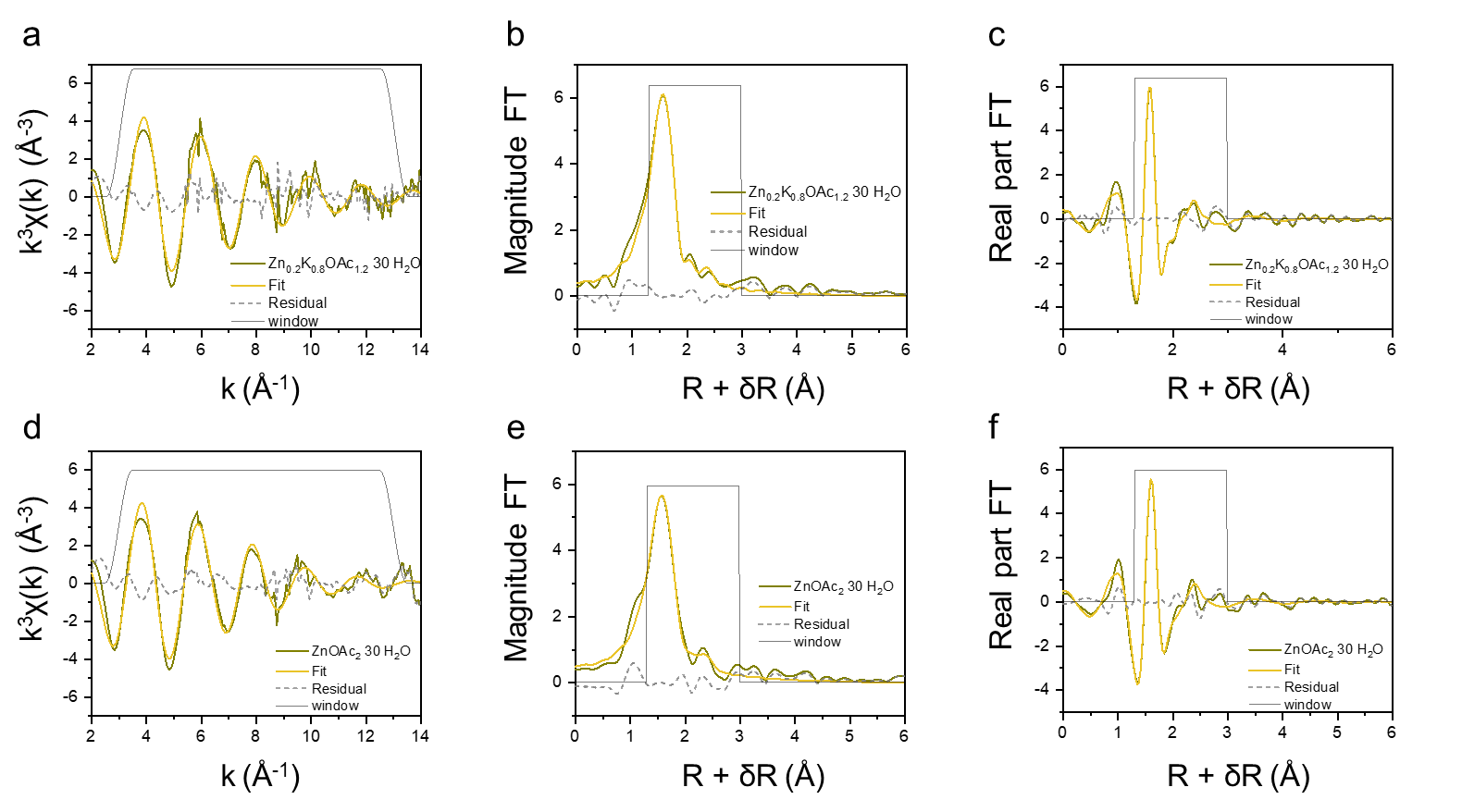


## **Figure S5.** Fitting to Zn(OAc)_4_ and Zn(H_2_O)_6_ clusters for the electrolyte a-c) X_Zn_=0.2 and d-f) X_Zn_=1.

**
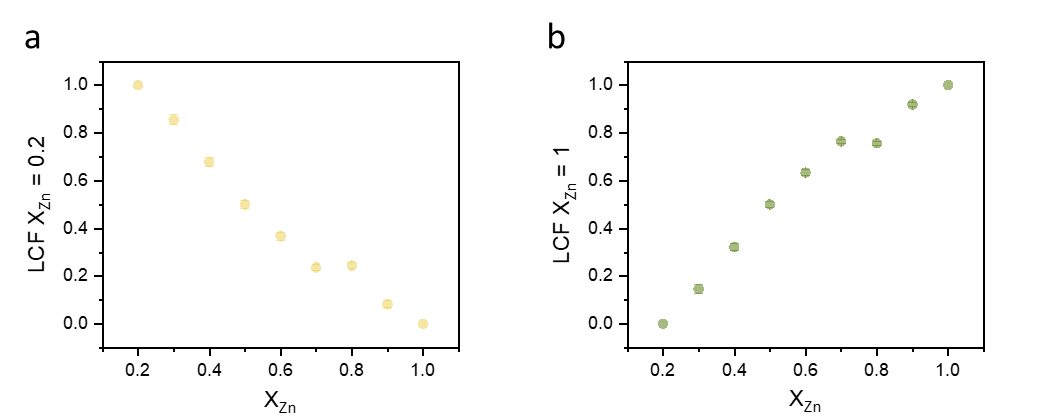
**

## **Figure S6.** Linear combination fitting for XAS as function of electrolyte composition a) fitting to X_Zn_=0.2 b) fitting to X_Zn_=1.


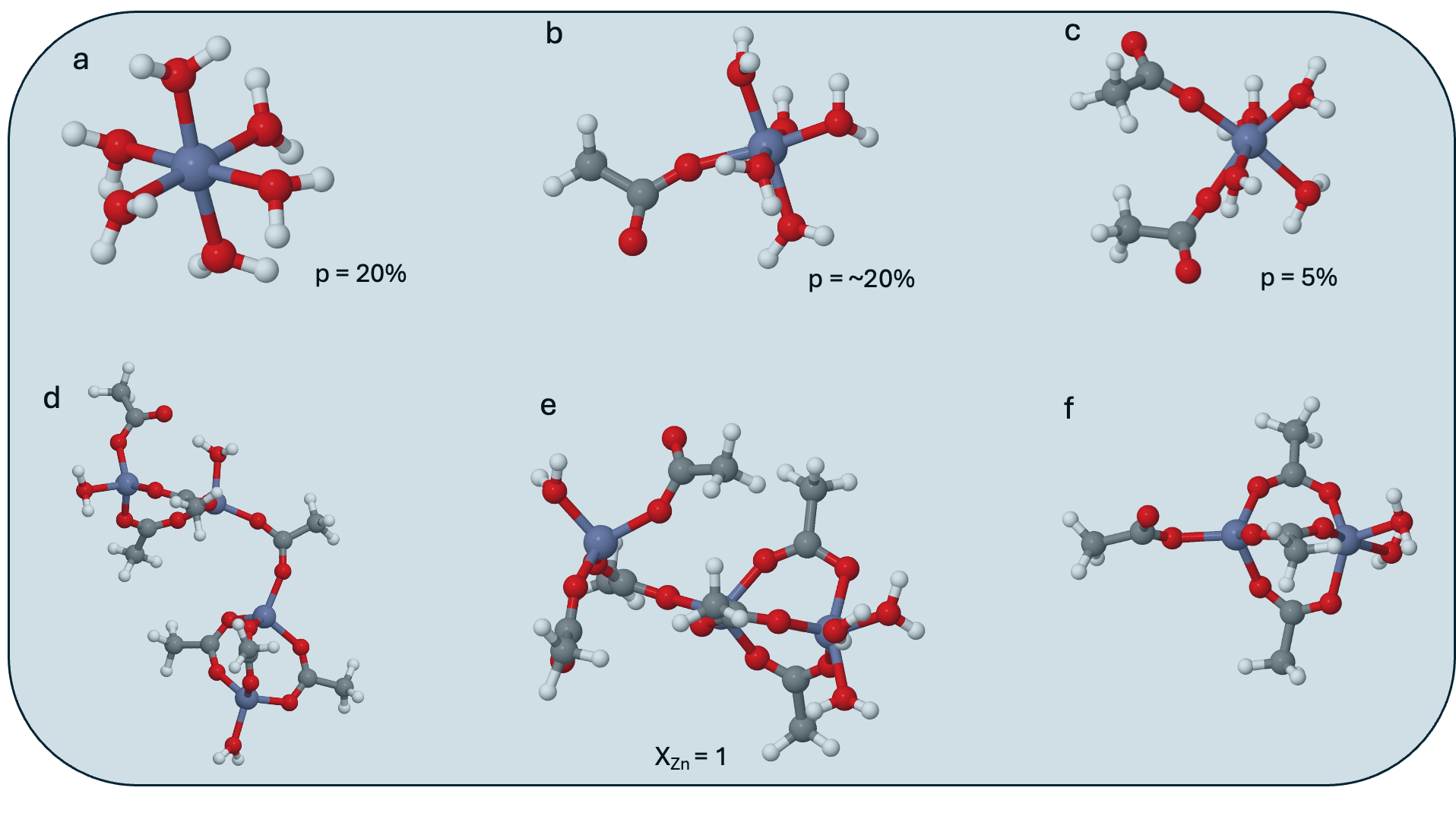


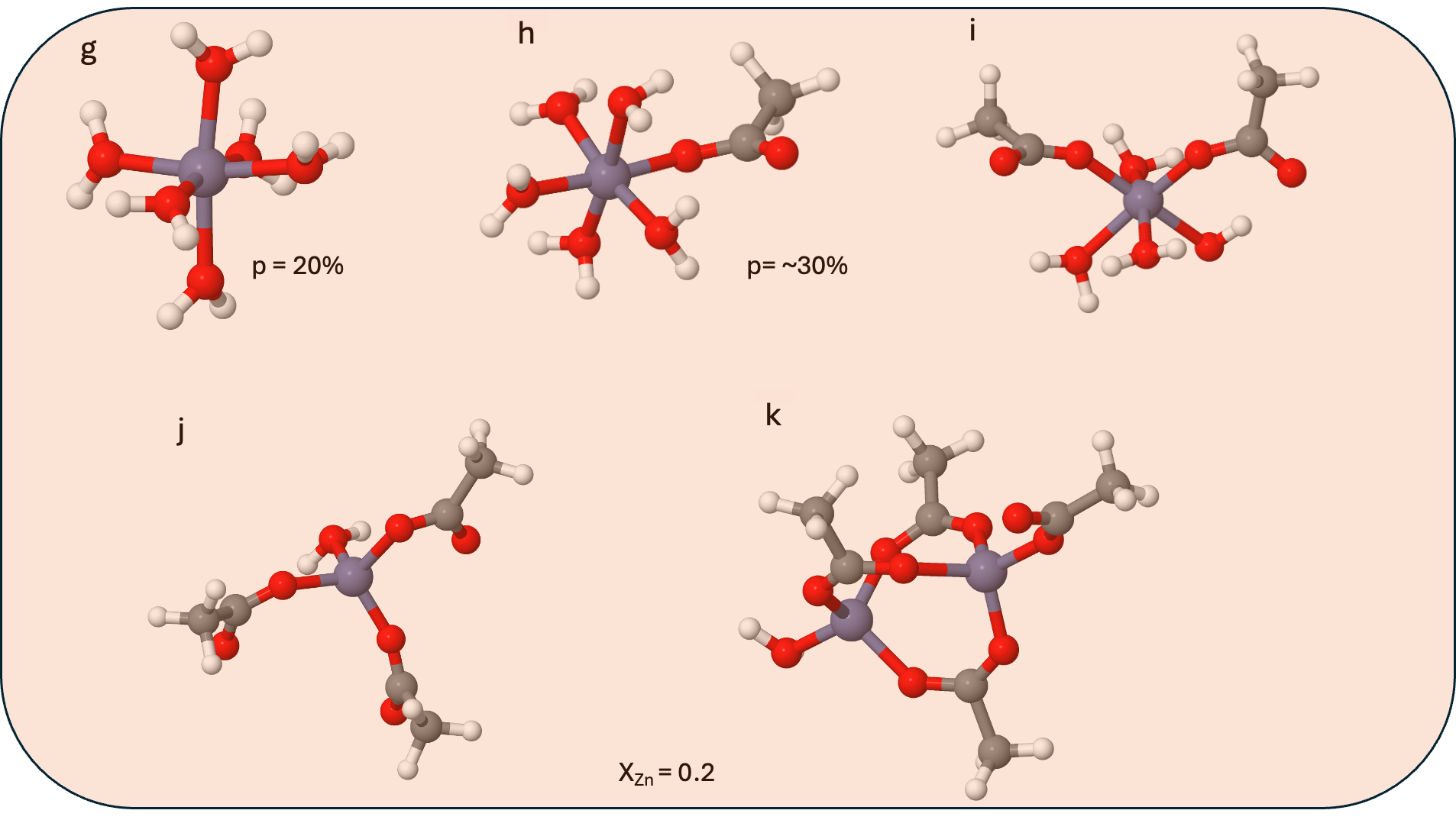


## **Figure S7.** Representative Zn solvates from MD simulations for X_Zn_ = 1.0 (a-f) and X_Zn_ = 0.2 (g-k). Color scheme: purple – Zn, red – O, grey – C, white – H.


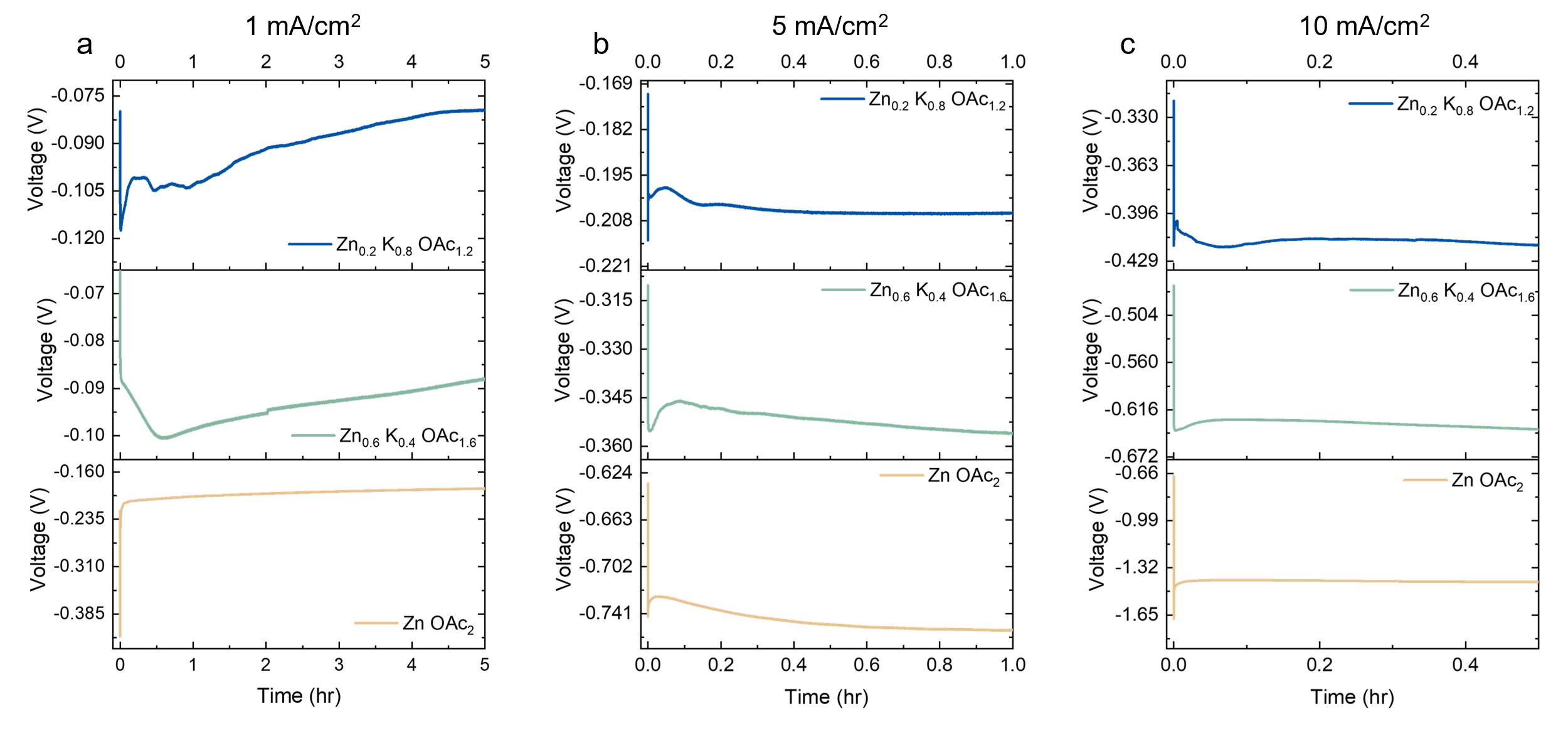


## **Figure S8.** Plating profiles for the SEM visualizations as function of electrolyte composition Zn_X_K_1-X_(OAc)_1+X_ ·30H_2_O X_Zn_=0.2, 0.6 and 1. a) results at 1 mA/cm^2^ b) results at 5 mA/cm^2^ c) results at 10 mA/cm^2^.


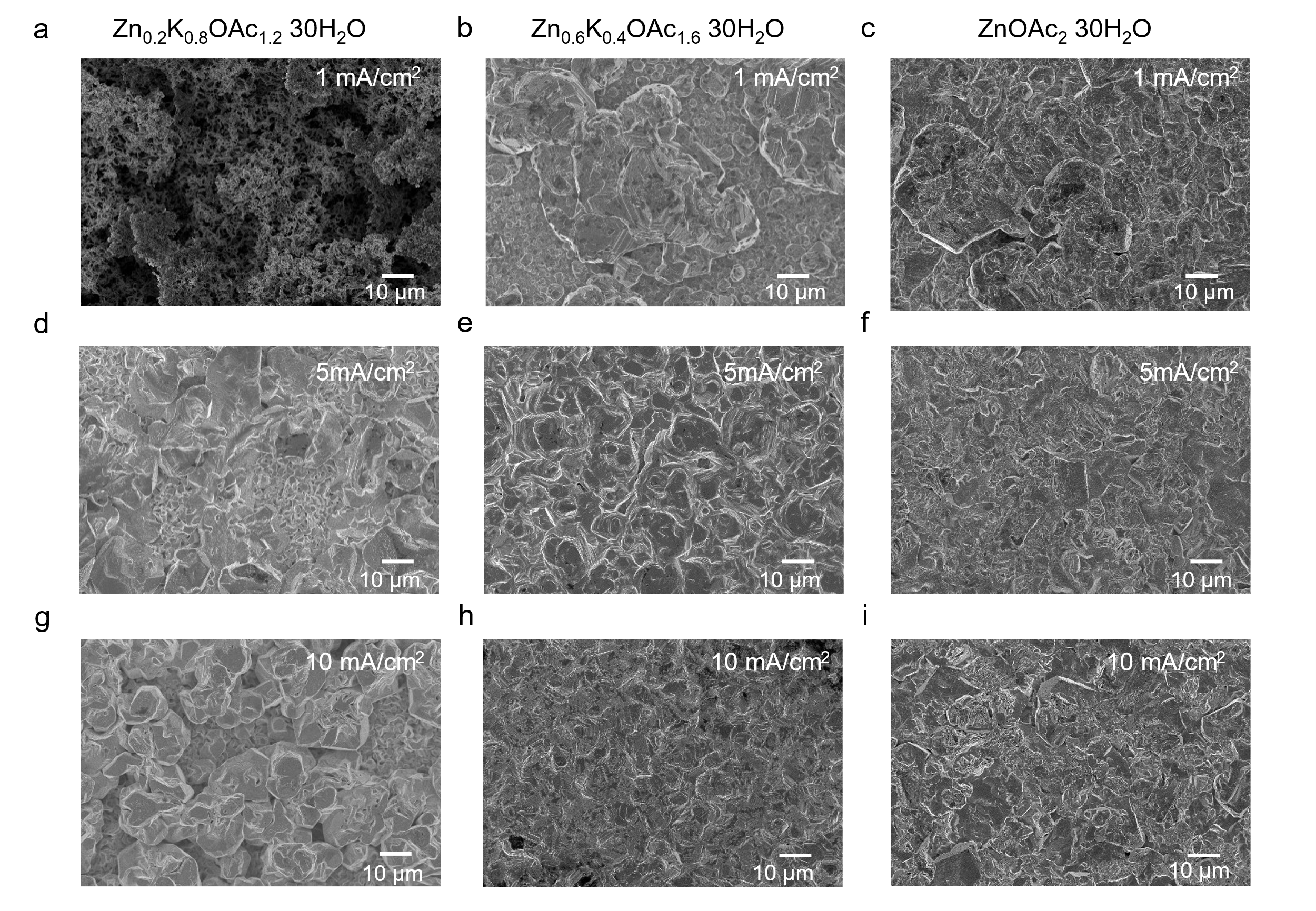


## **Figure S9.** Top-view SEM visualization for the plating of 5 mAh/cm^2^ on Si/Zn substrates (200nm Zn) at different current densities for the electrolytes Zn_X_K_1-X_(OAc)_1+X_ ·30H_2_O, for 0.2<X_Zn_<1. **a-c)** Deposition current of 1 mA/cm^2^. **d-f)** Deposition current of 5 mA/cm^2^. **g-i)** Deposition current of 10 mA/cm^2^. The columns correspond to the electrolyte composition.


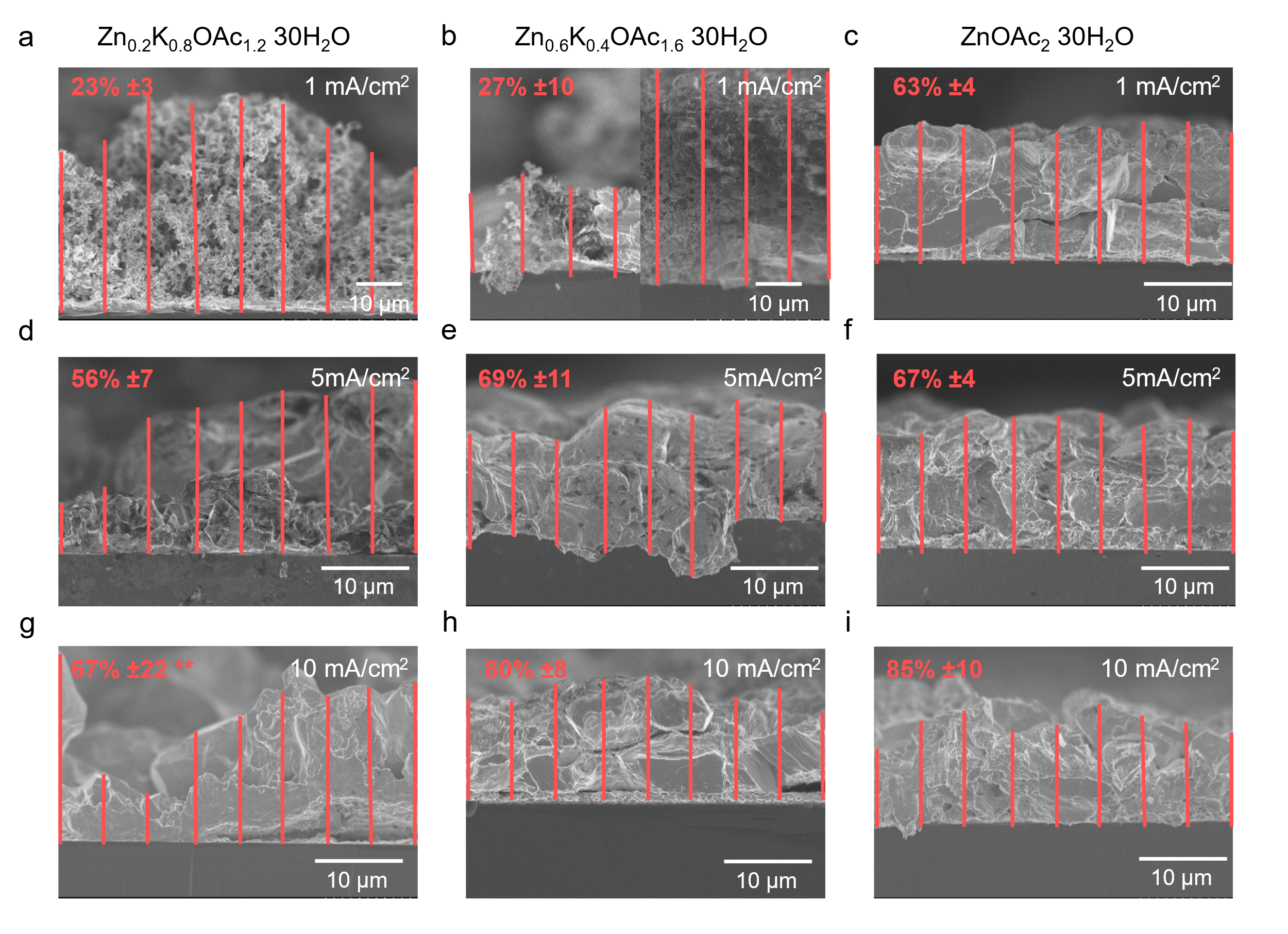


## **Figure S10.** Transversal SEM visualization with Zn plating density quantification for the plating of 5 mAh/cm^2^ (8.4 µm) on Si/Zn substrates (200 nm Zn) at different current densities for the electrolytes Zn_X_K_1-X_(OAc)_1+X_ ·30H_2_O, for 0.2<X_Zn_<1. **a-c)** Deposition current of 1 mA/cm^2^. **d-f)** Deposition current of 5 mA/cm^2^. **g-i)** Deposition current of 10 mA/cm^2^. The columns correspond to the electrolyte composition.


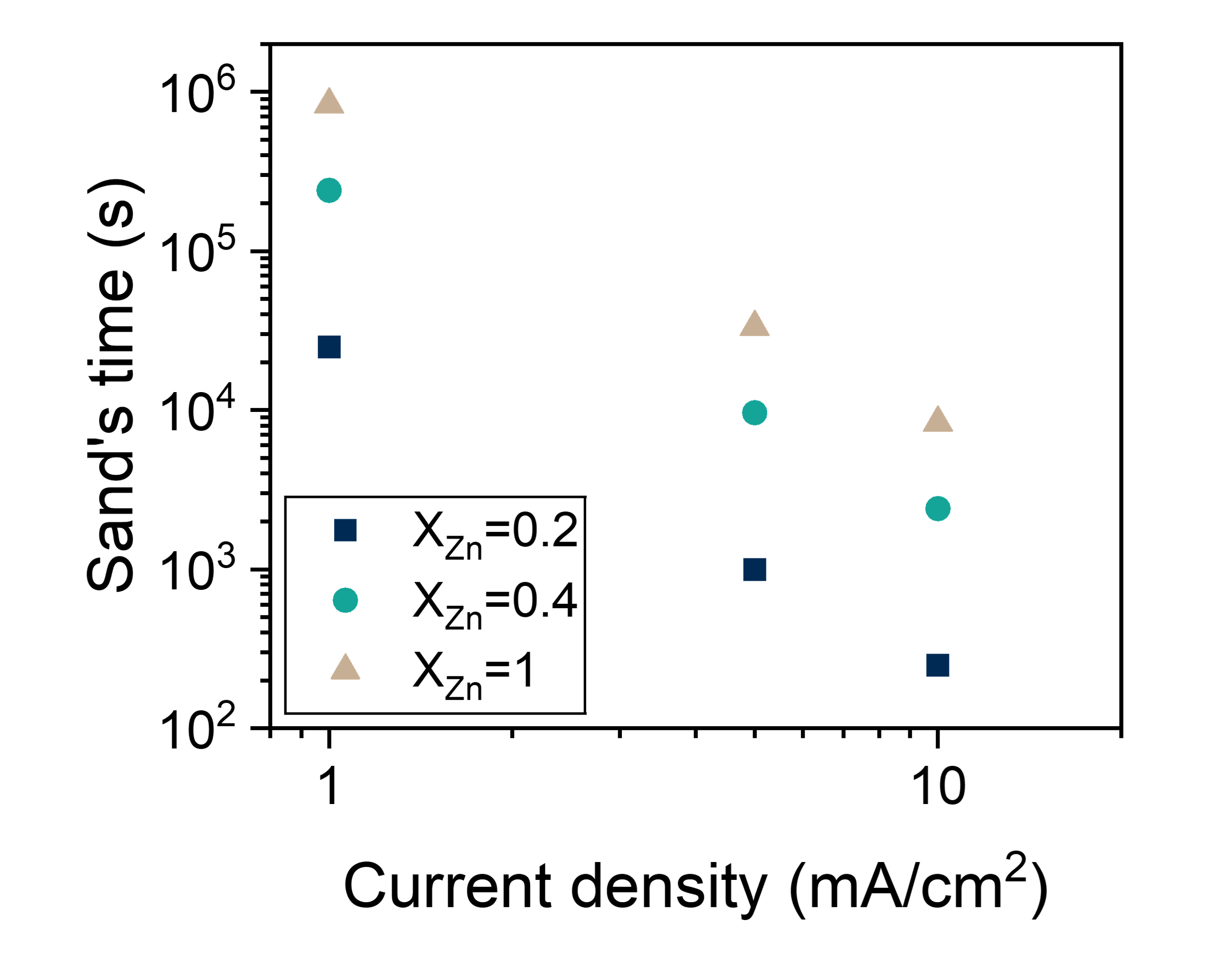


## **Figure S11.** Sand’s time as function of deposition current and electrolyte composition for the electrolytes Zn_X_K_1-X_(OAc)_1+X_ ·30H_2_O, with X_Zn_=0.2, 0.6 and 1.


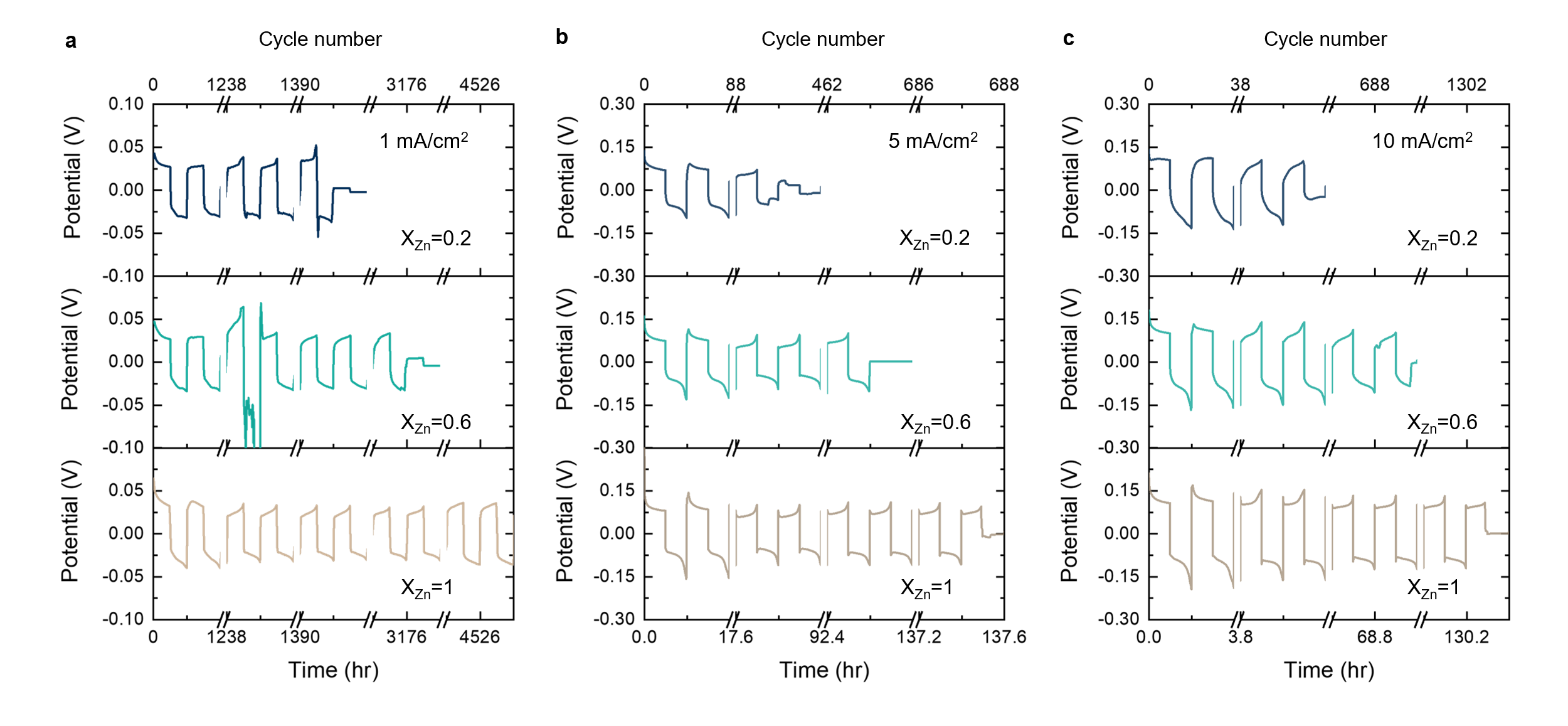


## **Figure S12.** Symmetric cycling zoom in depicting the first cycles and the moment of short-circuit for each cell as function of electrolyte composition. a-c. Show the cycling at currents of 1, 5 and 10 mA/cm^2^ for a charge of 0.5 mAh/cm^2^ respectively.


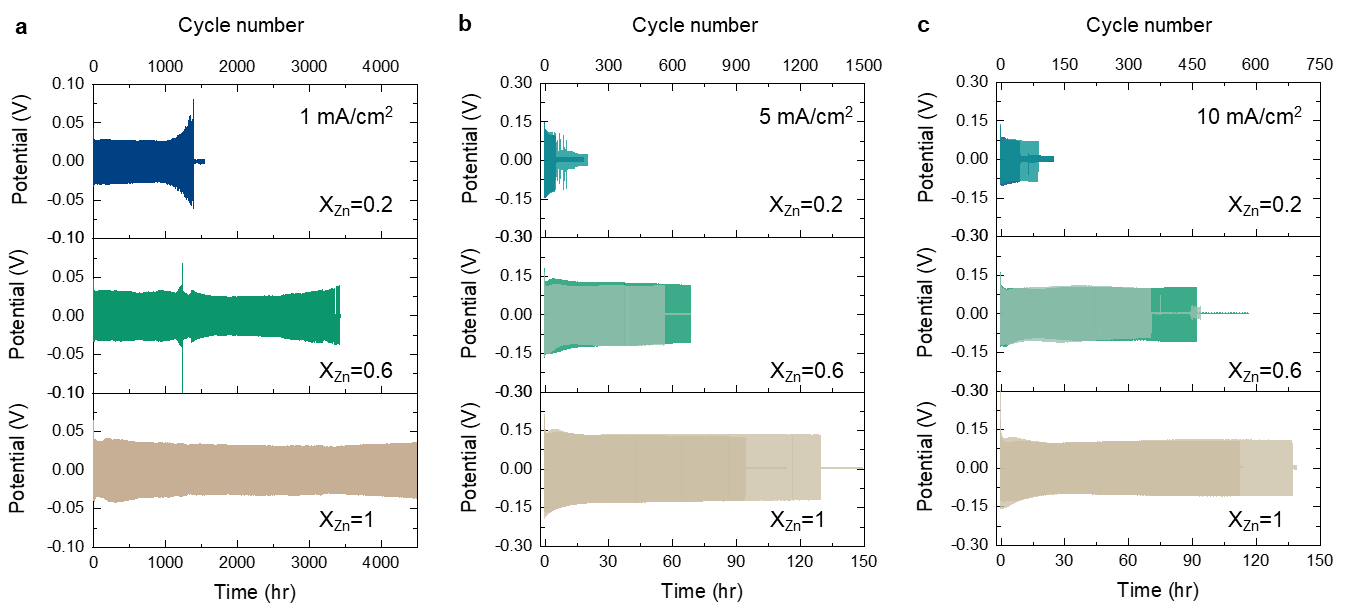


## **Figure S13.** Symmetric cycling duplicates depicting reproducibility and cell to cell variation. a-c. Show the cycling at currents of 1, 5 and 10 mA/cm^2^ for a charge of 0.5 mAh/cm^2^ respectively.


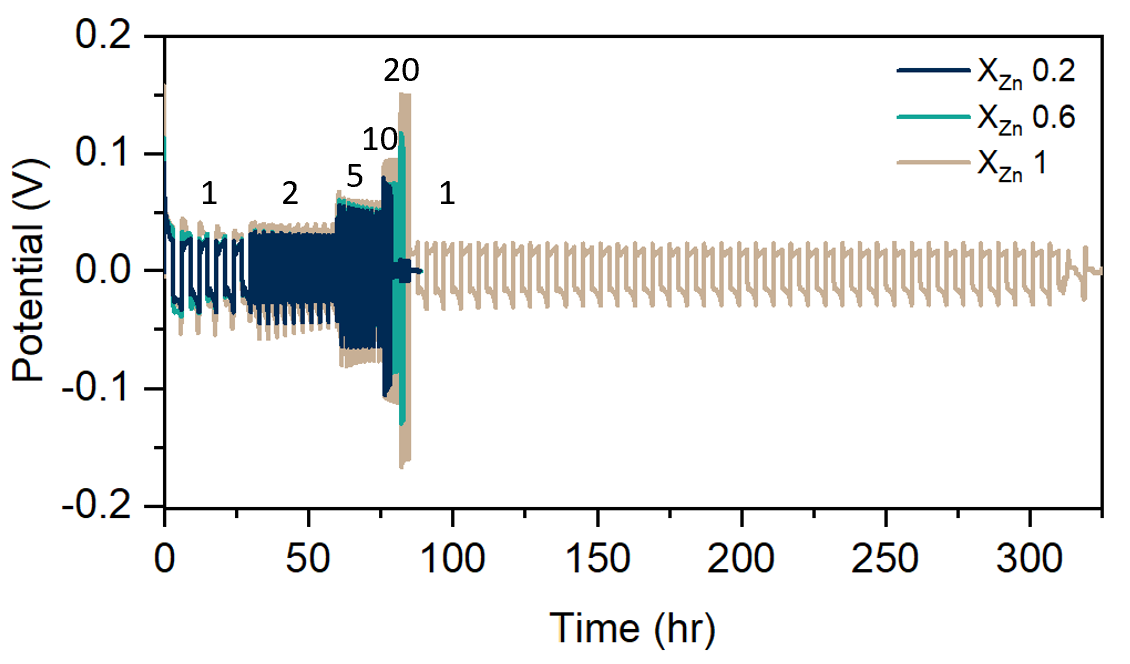


## **Figure S14.** Rate capability tests for electrolytes with X_Zn_ = 0.2, 0.6, 1. The cells were cycled to the capacity of 3 mAh/cm^2^ at current densities between 1 and 20 mA/cm^2^.


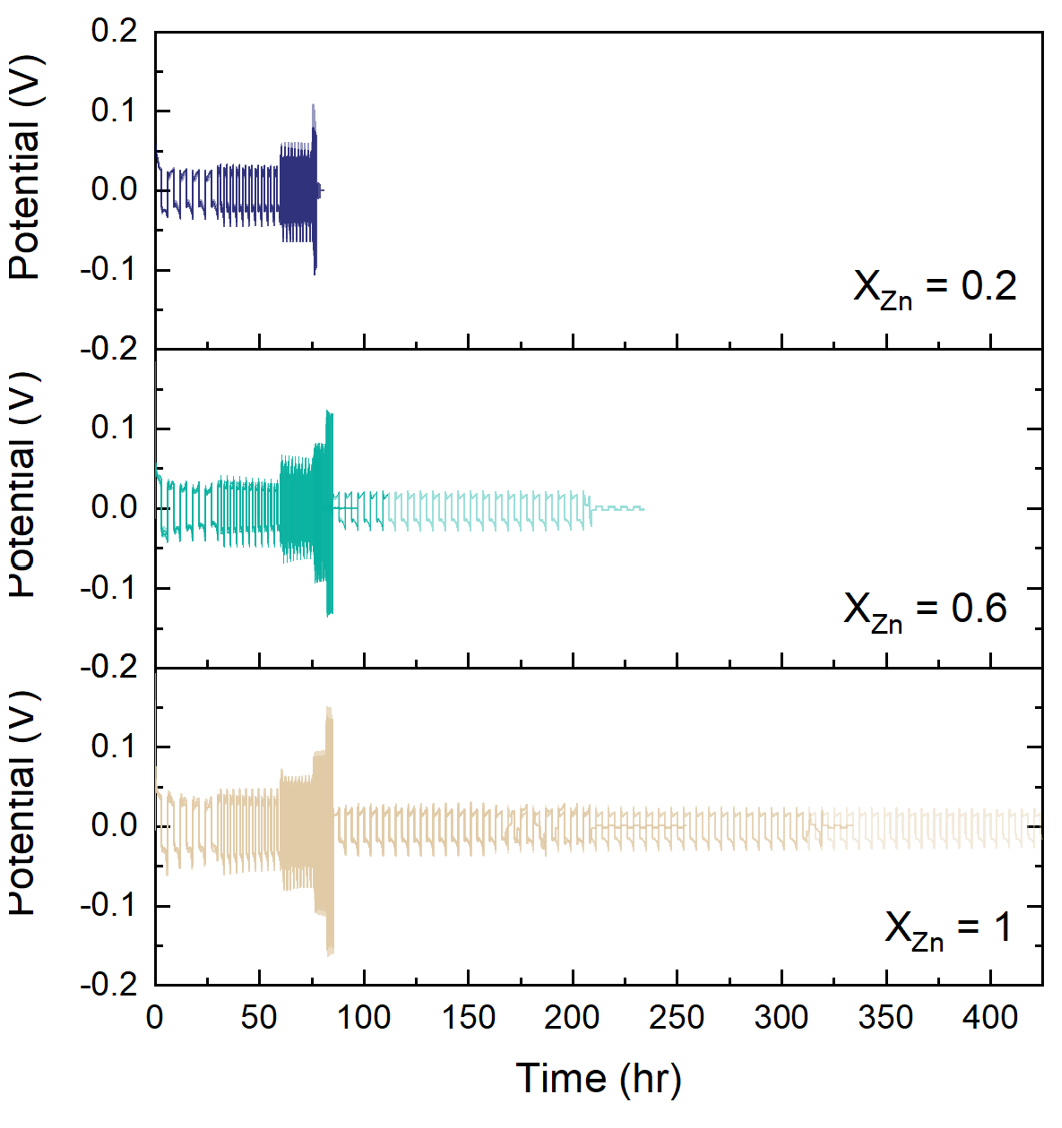


## **Figure S15.** Repetitions of the rate capability tests for electrolytes with X_Zn_ = 0.2 (n=3), 0.6 (n=4), 1 (n=4), where n is the number of test repetitions. The cells were cycled to the capacity of 3 mAh/cm^2^ at current densities between 1 and 20 mA/cm^2^.
